# Comprehensive expression analysis suggests overlapping and specific roles of rice glutathione S-transferase genes during development and stress responses

**DOI:** 10.1186/1471-2164-11-73

**Published:** 2010-01-29

**Authors:** Mukesh Jain, Challa Ghanashyam, Annapurna Bhattacharjee

**Affiliations:** 1 National Institute of Plant Genome Research (NIPGR), Aruna Asaf Ali Marg, New Delhi - 110 067, India

## Abstract

**Background:**

Glutathione S-transferases (GSTs) are the ubiquitous enzymes that play a key role in cellular detoxification. Although several GSTs have been identified and characterized in various plant species, the knowledge about their role in developmental processes and response to various stimuli is still very limited. In this study, we report genome-wide identification, characterization and comprehensive expression analysis of members of GST gene family in crop plant rice, to reveal their function(s).

**Results:**

A systematic analysis revealed the presence of at least 79 GST genes in the rice genome. Phylogenetic analysis grouped GST proteins into seven classes. Sequence analysis together with the organization of putative motifs indicated the potential diverse functions of GST gene family members in rice. The tandem gene duplications have contributed a major role in expansion of this gene family. Microarray data analysis revealed tissue-/organ- and developmental stage-specific expression patterns of several rice GST genes. At least 31 GST genes showed response to plant hormones auxin and cytokinin. Furthermore, expression analysis showed the differential expression of quite a large number of GST genes during various abiotic stress (20), arsenate stress (32) and biotic stress (48) conditions. Many of the GST genes were commonly regulated by developmental processes, hormones, abiotic and biotic stresses.

**Conclusion:**

The transcript profiling suggests overlapping and specific role(s) of GSTs during various stages of development in rice. Further, the study provides evidence for the role of GSTs in mediating crosstalk between various stress and hormone response pathways and represents a very useful resource for functional analysis of selected members of this family in rice.

## Background

Glutathione transferases (GSTs, EC 2.5.1.18), formerly known as glutathione S-transferases, are the enzymes involved in cellular detoxification by conjugating the tripeptide (γ-Glu-Cys-Gly) glutathione (GSH) to a wide variety of substrates such as endobiotic and xenobiotic compounds [1]. GSTs have been identified in all the organisms, including plants, animals, fungi and bacteria analyzed to date [2,3]. Although most of GSTs exist as soluble enzymes, distantly related mitochondrial Kappa GSTs and microsomal GSTs have also been identified in animals [4]. GST proteins are represented by a multi-gene family in plants similar to other organisms [5-7]. Several GSTs have been identified and characterized in various plant species with differential and overlapping substrate specificities [5,8,9]. Based on the predicted amino acid sequences, the soluble GSTs in plants have been grouped into several classes, including Phi, Tau, Lambda, dehydroascorbate reductase (DHAR), Theta, Zeta, elongation factor 1 gamma (EF1G) and tetrachlorohydroquinone dehalogenase (TCHQD). Among these classes, Phi, Tau, Lambda and DHAR classes are plant specific [10].

Plant GSTs have been a focus of attention because of their role in herbicide detoxification. Some evidences showed that GSTs are present at every stage of plant development from early embryogenesis to senescence and in every tissue type examined [5,7,11]. GSTs have been found to be differentially regulated by a variety of stimuli, including abiotic and biotic stresses, plant hormones such as auxins, cytokinins and ABA, heavy metals, GSH and hydrogen peroxide [12-15]. Despite their suspected crucial role in stress responses and significant efforts made, the specific role(s) of GST enzymes have not been elucidated. The role of plant GSTs has also been proposed in the transport and metabolism of secondary compounds [16-18]. Plant GSTs can also act as glutathione peroxidases [19,20], protect cells from oxygen toxicity [21] and suppress apoptosis [22]. Some of plant GSTs were originally identified as auxin- and cytokinin-binding proteins [23-25], pointing their role in hormone signal transduction pathways as well.

Among GSTs, the members of Tau and Phi classes are most studied in plants. The reason may be attributed to their larger number. They are dimeric and catalyze the conjugation of a diverse range of xenobiotics and detoxify selective herbicides [10]. Theta GSTs have limited transferase activity towards xenobiotics but are highly active GSH-dependent peroxidases [10]. Zeta class GSTs have been shown to differ from other GSTs in showing no GSH conjugating or GSH peroxidase activity, rather these are involved in GSH-dependent tyrosine catabolism [26,27]. The recently discovered DHAR class GSTs are monomeric and act as GSH-dependent oxidoreductases [8]. EF1G class of GST proteins encodes γ subunit of eukaryotic translation elongation factor. It has been proposed that the N-terminal GST domain present in this class of proteins may be involved in mediating the assembly of EF1 and regulation of formation of multisubunit complexes containing EF1 [28]. Another recently identified GST from *Arabidopsis *closely resembles the TCHQD enzymes from prokaryotes [10]. The functional characterization of this protein has not been reported.

Although quite a few GST proteins have been characterized in rice, the functions of majority of members in this family remain unknown. In the first identification and classification of GST family in rice based on analysis of EST database and unfinished genomic sequence, a total of 61 members were identified and their expression patterns analyzed by querying EST databases [7]. In this study, the members of GST family in rice have been reanalyzed based on complete genome sequence and annotation, and a total of 79 putative GST genes were identified. In addition, a comprehensive expression analysis during various stages of development, hormone treatments, abiotic and biotic stress conditions, have been performed. The results reported in this study will provide a very useful reference for further functional analysis of members of this family in rice.

## Results and Discussion

### Identification of genes encoding GST proteins in rice

GSTs are represented by multigene family in plants. Several members of GST family have been identified and divided into different classes in *Arabidopsis*, maize and soybean [5,6]. Soranzo et al [7] reported the presence of 61 members of GST family in rice based on analysis of EST database and unfinished genomic sequence and divided them into four classes. In this study, we performed domain search for the proteins containing GST N-terminal domain (PFAM domain PF02798) in the Rice Genome Annotation Project (RGAP) database (release 5) or HMM profile search against the downloaded proteome of rice. Subsequently, the proteins which did not show the presence of GST_N domain (characteristic of GST proteins) in SMART analysis were eliminated. Taken together, a total of 79 non-redundant gene loci were predicted to contain GST_N domain and encode putative GST proteins in rice. The number of GST proteins predicted in this study is 29.5% greater than the previously reported number in rice (61) [7]. A comparative analysis showed that 52 among the 61 ESTs/cDNA clones encoding GST proteins in rice reported by Soranzo et al [7] correspond to the gene loci predicted in this study. The sequences for five genes (NM_1903851, *OsGSTF11*; P0493G01.11, *OsGSTF13*; OJ1006_F06.6, *OsGSTF16*; OSJNBa0033P04.19, *OsGSTU36 *and OSJNBa0018H01.7, *OsGSTMU37*) could not be retrieved as they have become obsolete entries in NCBI, *OsGSTU6 *(AF379376) represents a viral sequence, *OsGSTT2 *(AY541762) has been annotated as a ty1-copia subclass retrotransposon, *OsGST35 *(AY533125) did not show the presence of GST_N domain and *OsGSTU7 *(AF402794) was redundant with another GST gene *OsGSTU18 *(AF402805). In total, this study reports 27 new gene loci encoding GST proteins in rice as compared to Soranzo et al [7]. The locus ID, open reading frame length, protein length and chromosomal location of all the 79 GST genes are given in Additional file 1.

To reveal the evolutionary relationship among the rice GST proteins, a phylogenetic tree was generated using their full-length protein sequences (Additional file 2). The results suggested that the rice GST family can be classified into seven classes (Additional file 2). Based on the protein sequence alignments and evolutionary relationship, largest number of GST genes (52) were included in Tau class followed by 17 genes in Phi, four in Zeta, two in DHAR, two in EF1G and one each in Theta and TCHQD classes. The members included in Tau, Phi, Zeta, DHAR, EF1G, Theta and TCHQD classes were designated as *OsGSTU*, *OsGSTF*, *OsGSTZ*, *OsDHAR*, *OsEF1G*, *OsGSTT *and *OsTCHQD*, respectively, followed by a number (Additional file 1). To keep the nomenclature of GST family consistent, the name of reported genes has been retained and the systematic names of the genes reported previously but not identified in this study were assigned to newly identified members (Additional file 1). All the newly identified members within a class were named according to their sequential position on rice chromosomes from top to bottom.

**Figure 1:**
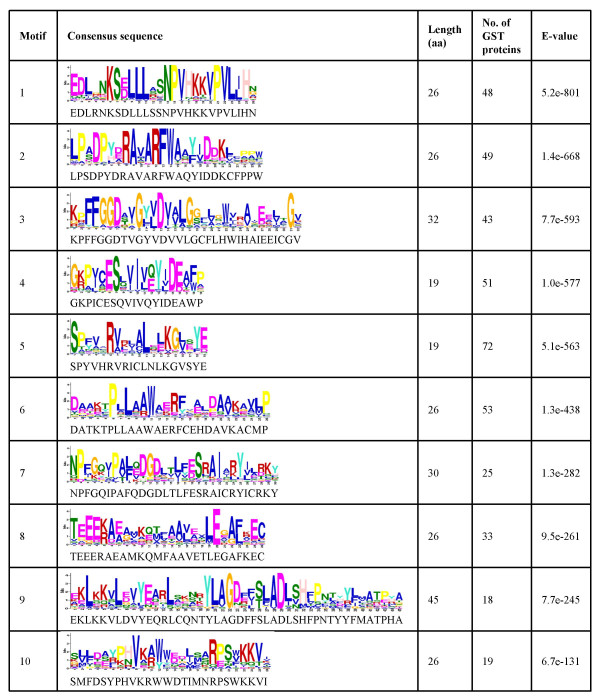
**Putative motifs predicted in rice GST proteins**. Significant motifs (e-value <e-100) of more than 10 amino acid length present in at least 10 GST proteins were predicted by MEME search. The consensus sequence, length (amino acids), number of GST proteins containing the motif and e-value of each predicted motif is given. The first residue in motif 5 represents the active site serine residue.

The pairwise comparison of GST protein sequences showed considerable sequence diversity with overall identity ranging from 8% to 92%. However, relatively high identities were found among the members of same class, for example, the sequence identity among the members of Tau class varied from 27% to 92% and for Phi class members from 24% to 84%. The multiple sequence alignments of full-length GST protein sequences showed that N-terminal is highly conserved, which contains active site serine residue fundamental to GST activity and GSH-binding site. The active site serine residue (position 15 in OsGSTU1) was conserved in all but 10 of the GST proteins. OsGSTU45, U47, U52, F11, F13, T1, DHAR1, DHAR2, EF1G1 and EF1G2 lack essential active site serine residue. An arginine residue (position 20 in OsGSTU1) was also conserved in most of GST proteins. In addition, serine residue (position 69 in OsGSTU1) in the GSH-binding domain was essentially conserved in all the GST proteins except OsGSTF9 and OsTCHQD1. The additional putative conserved motifs in GST proteins were investigated using Multiple Em (Expectation Maximization) for Motif Elicitation (MEME) program. We identified ten highly significant (<e-100) putative conserved motifs of more than 10 amino acids in length present in at least ten of the GST proteins (Fig. 1, Additional file 3). The motifs 1, 4, 5 and 7 formed a part of GST N-terminal domain. Among the ten predicted putative motifs, some were specific to the members of a particular class and others were conserved in two to many classes. Motifs 1, 2, 3 and 8 were specific to Tau class only; motif 4 was present in members of Tau and DHAR class and motif 6 in Tau and Zeta class members. Motif 5, which harbors the active site serine residue, was present in most of members of Tau, Phi, Zeta and TCHQD classes. The members of Phi, Zeta, Theta, EF1G and TCHQD harbors motif 7. Motif 9 was present in Phi, EF1G and TCHQD classes, whereas motif 10 was present in members of Phi, EF1G and DHAR classes. These putative conserved motifs may provide diversity in functions of the GST proteins.

### Tandem duplications are responsible for the family expansion

**Figure 2:**
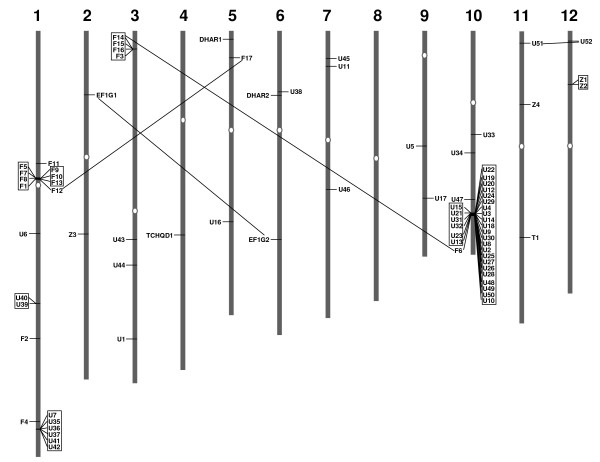
**Chromosomal distribution of rice GST genes**. The chromosome number is indicated at the top of each chromosome. GST genes localized on duplicated chromosomal segments are connected by lines. The genes labeled on left side are oriented from bottom to top and those on right side are oriented from top to bottom on rice chromosomes. Six clusters (three on chromosome 1 and one each on chromosome 1, 10 and 12) of tandemly arranged GST genes are indicated in boxes. Exact position of each GST gene on rice chromosome pseudomolecules is given in Additional file 1.

The rice genome has undergone several rounds of genome-wide duplication events, including polyploidy, which has great impact on the expansion of a gene family in the genome. To investigate the contribution of gene duplication in the expansion of GST gene family, the chromosomal location of each GST gene was determined based on the information provided by RGAP (Additional file 1). The chromosomal localization of all members of this family indicated a non-random distribution (Fig. 2). Chromosome 8 was devoid of GST genes whereas chromosome 10 encoded highest number (32 of 79, 40.5%) of the GST family members followed by chromosome 1 (20 of 79, 25.3%). A total of six clusters of tandemly arranged GST genes were observed on different chromosomes. Chromosome 1 harbors three clusters of GST genes, first cluster of seven members of Phi class, second cluster of two members of Tau class and third cluster of six members of Tau class. The fourth cluster of 4 members of Phi class was present on chromosome 3. The fifth and largest cluster localized on chromosome 10 contains 28 Tau class GST genes present in tandem at a single locus. The two Zeta class GST genes formed the sixth cluster on chromosome 12. This data suggested the major contribution of tandem duplications (51 of 79, 64.6%) to the GST family expansion. On the other hand, we found eight members of GST family located on the segmentally duplicated regions. Since the number of GST genes located on segmentally duplicated regions is much smaller than those present in tandem, the localized gene duplications appear to have contributed a major role in expansion of this gene family.

**Figure 3:**
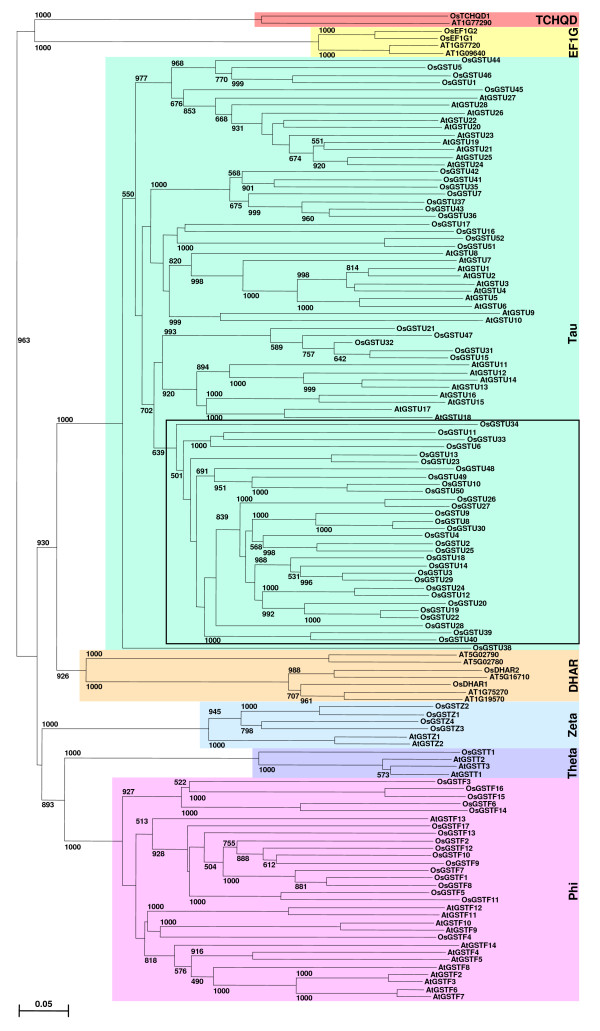
**Phylogenetic relationship among rice and *Arabidopsis *GST proteins and their classification**. The unrooted tree was constructed based on multiple sequence alignment of full-length protein sequences using ClustalX program by neighbor-joining method with 1000 bootstrap replicates. The numbers at the nodes represent bootstrap values (≥ 500) from 1000 replicates. All the rice and *Arabidopsis *GST proteins grouped into seven classes. The bar indicates 0.1 substitutions per site. The cluster of Tau class GST genes present only in rice is indicated in box.

Rice genome encodes significantly higher (79) number of GST genes as compared to *Arabidopsis *(54). To explore the expansion of GST gene family members in rice vis-à-vis *Arabidopsis*, a phylogenetic tree was constructed based on the multiple sequence alignment of their full-length protein sequences (Fig. 3). The phylogenetic tree showed that GST proteins from rice and *Arabidopsis *belonging to the same class were clustered together. In addition, GST proteins of rice and *Arabidopsis *within a class were usually clustered in species-specific manner. This indicates that GST proteins of each class existed before the divergence of monocots and dicots and later on expanded independently in species-specific manner. This type of species-specific expansion of other gene families has been observed as well [29,30]. Furthermore, the degree of expansion of Tau class varied significantly in rice and *Arabidopsis*. Only 28 members of Tau class have been predicted in *Arabidopsis*, whereas this class is comprised of 52 members in rice. It seems tandem duplication events have contributed significantly towards evolution of Tau class GST genes in rice. The analysis of phylogenetic tree revealed that a major group of GST proteins comprised of 30 proteins within Tau class present in rice was absent in *Arabidopsis *(Fig. 3). It may be speculated that these GST proteins were lost in *Arabidopsis *or evolved in rice after divergence of monocots and dicots and may perform monocot specific functions. Likewise, the expansion of Phi, Zeta and DHAR classes is more in rice as compared to *Arabidopsis*. Although the Zeta and Theta class GSTs have been suggested to represent ancestral genes, they are poorly represented in plants [14]. Surprisingly, on the other hand, the presence of plant-specific Phi and Tau class GSTs is more numerous. This could be explained due to the rapid evolution of Phi and Tau class GSTs in plants, which may perform diverse plant-specific functions.

### Differential expression of GST gene family members in various tissues/organs

Although the roles of GSTs have been explored in various stress responses, the evidences for their role in plant growth and development are very limited. The overlapping and tissue-specific expression patterns of GST genes have been observed in some plant species, including rice, by querying EST databases [5,7,9]. The study of gene expression patterns of all the members of a gene family provides insight into their functional diversification. The expression evidence for 62 of the rice GST genes was found in terms of the availability of their corresponding full-length cDNA and/or EST evidence. We surveyed the transcript accumulation of GST genes across a wide range of tissues/organs and developmental stages of rice employing two approaches. In the first approach, we used the data from rice Massively Parallel Signature Sequencing (MPSS) database to quantify the expression of individual GST gene. MPSS technology provides a quantitative measure of transcript accumulation of virtually all the genes in a tissue sample in terms of number of small signature sequences corresponding to each gene [31]. The survey of 22 rice MPSS libraries [32] representing 18 tissue samples showed that at least 77 GST genes have corresponding 17 base signatures, suggesting that most of the GST genes are expressed in rice. However, significant signatures (that uniquely identify individual GST gene) were found for 61 GST genes (Additional file 4). The number of tags (in tpm, tags per million) for rice GST genes varied significantly, indicating marginal (1-3 tpm) to strong (>250) expression. In addition, the number of tags revealed differential expression patterns of individual GST genes in various rice tissues/organs.

**Figure 4:**
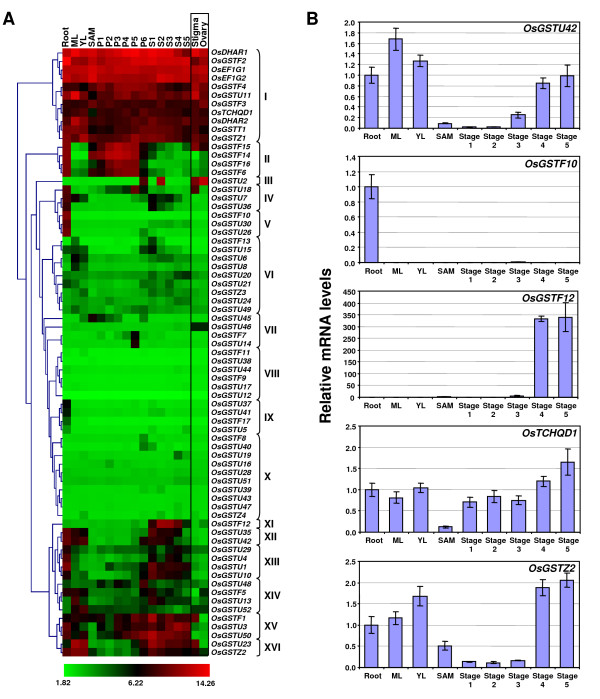
**Expression patterns of rice GST genes in various tissues/organs and developmental stages**. (A) Hierarchical clustering analysis of 71 GST genes represented on Affymetrix Rice Genome Array is shown. For clustering we used average log signal values for three biological replicates of each sample after normalization of the raw data (Additional file 6). The color scale for log signal values is shown at the bottom. Clusters (I-XVI) are marked on the right. ML, mature leaf; YL, Y-leaf; SAM, shoot apical meristem; P1-P6, stages of panicle development; S1-S5, stages of seed development. (B) Real-time PCR analysis of selected genes to validate their differential expression during various stages of development. The mRNA levels for each gene in different tissue samples were calculated relative to its expression in root. The error bars represent standard deviation. ML, mature leaf; YL, Y-leaf; SAM, shoot apical meristem; Stage 1-3 represent stages of panicle development (Stage 1 corresponds to P1 and P2; Stage 2, P3 and P4; Stage 3, P5 and P6); Stage 4 and 5 represent stages of seed development (Stage 4 corresponds to S1 and S2; Stage 5, S3-S5).

Microarray represents a high throughput means to analyze the gene expression of all the members of a gene family and to identify genes involved in a particular biological process. In the second approach, we used the microarray data for various tissues/organs and developmental stages available at GEO database under the accession numbers GSE6893 and GSE7951. The series GSE6893 includes microarray data from 45 hybridizations representing three biological replicates each of 15 different tissues/organs and developmental stages [30], whereas series GSE7951 includes the microarray data from 12 hybridizations representing 9 different tissue samples [33]. Because three biological replicates were available only for stigma and ovary in the series GSE7951 dataset, only these data were used in this analysis. All the tissues/organs and developmental stages for which microarray data was analyzed in this study are summarized in Additional file 5. The probe sets representing GST genes on the Affymetrix array were identified using Rice Multiplatform Microarray Search Tool. A total of 71 GST genes were represented on the Affymetrix arrays. There was no probe set on the arrays corresponding to the remaining GST genes. The average log signal values of 71 GST genes were extracted after whole-chip data processing for all the tissue samples using Genespring GX software (Additional file 6). We used TIGR Multi Experiment Viewer to perform hierarchical clustering analysis of the expression of 71 GST genes based on their average log signal values (Fig. 4A). Distinct transcript abundance patterns of GST genes were readily identified in the microarray data analyzed. Many of the GST genes showed preferential accumulation of transcripts in a given tissue/organ or developmental stage. We grouped GST genes roughly in 16 clusters (cluster I-XVI) with similar expression patterns (Fig. 4A). The number of genes varied from one (cluster III and XI) to 11 (cluster I) in these clusters. Cluster I include 11 GST genes which are highly expressed and cluster VIII include six very lowly expressed GST genes in all the tissues/organs or developmental stage analyzed. Ten genes included in cluster X also exhibited very low expression in all/most of the tissues analyzed. The GST genes which showed very low expression in various tissues and/or developmental stages analyzed might express in specific cell-type(s) or tissue/condition other than those included in this study. Other reason for detection of very low expression of these GST genes may be attributed to the improper/hampered hybridization of their fragmented RNA molecules to the oligos represented on the chip because of the presence of specific single nucleotide polymorphisms in the rice varieties used for microarray experiments. However, other GST genes exhibited transcript abundance in one or more distinct tissue/organ or developmental stage analyzed. The expression of few GST genes was restricted to a particular tissue/organ/developmental stage, whereas other GST genes exhibited preferential expression in narrow to wide window of developmental stages. Four genes in cluster II are expressed preferentially in root and stages of panicle development. Seven GST genes (*OsGSTF10*, *U26 *and *U30 *in cluster V at high level and *OsGSTF17*, *U5*, *U37 *and *U41 *in cluster IX at low level) are expressed exclusively in root. The expression of *OsGSTF12 *is restricted to seed development stages (S1-S5). Two genes from cluster VII, *OsGSTF7 *and *U14*, were preferentially expressed during P5 stage of panicle development and the expression of *OsGSTU46 *was restricted to stigma and ovary. Four genes (two each in clusters XII and XVI) are expressed preferentially in vegetative tissues, late stages of panicle development and seed development stages. These genes may perform specific roles in these tissues/organs or developmental stage. The validation of differential gene expression of selected GST genes in various tissues/organs and developmental stages by real-time PCR analysis showed very good agreement with the microarray results (Fig. 4B). These results suggest the involvement of GSTs in various developmental events in rice.

The expression of eight Tau class GST genes (*OsGSTU9*, *22*, *25*, *27*, *31*, *32*, *33 *and *34*) for which microarray data was not available, was explored in terms of availability of their corresponding FL-cDNA, EST and/or MPSS tags. We found all these evidences of expression (FL-cDNA, EST and MPSS tags) for three GST genes (*OsGSTU9*, *U27 *and *U34*) and one GST gene (*OsGSTU22*) had corresponding FL-cDNA/EST available. Although MPSS tags were available for other four GST genes, significant (that uniquely identify individual gene) tags were available for two (*OsGSTU31 *and *33*) of them. Among these GST genes, *OsGST9 *was expressed in a wide range of tissues, whereas *OsGSTU27*, *31*, *33 *and *34 *were preferentially expressed in stressed and/or non-stressed young roots (Additional file 4). Taken together, our results indicate that all the 79 GST genes identified in this study are expressed in one or the other rice tissue and exhibit overlapping and/or specific expression patterns with quantitative differences.

### Duplicated GST genes exhibit redundant and divergent expression patterns

**Figure 5:**
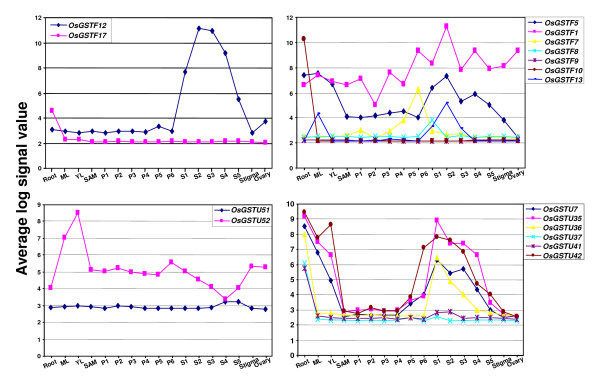
**Expression patterns of representative duplicated GST genes in various tissues/organs and developmental stages**. The average log signal values (from three biological replicates) for each gene in all the samples analyzed is presented on Y-axis. The expression patterns of all the duplicated GST genes in various tissues/organs and developmental stages is given in Additional file. ML, mature leaf; YL, Y-leaf; SAM, shoot apical meristem; P1-P6, stages of panicle development; S1-S5, stages of seed development.

As discussed above, we found quite a large number of duplicated GST genes in rice. Gene duplication raises the question about their functional redundancy and also serves as a mechanism to increase functional diversity. After duplication, genes may undergo diversification of gene function such as neo-functionalization, sub-functionalization, non-functionalization or hypo-functionalization [34-36]. The expression patterns of duplicated genes indicate their evolutionary fates. The functional diversification has been proposed to be important for the retention of duplicated genes. To reveal the functional redundancy/diversification of duplicated GST genes, their expression patterns were analyzed. Among the four GST gene pairs localized on duplicated chromosomal segments, two pairs *OsGSTF6*/*F14 *and *OsEF1G1*/*EF1G2 *exhibit similar expression patterns, whereas other two pairs *OsGSTF12*/*F17 *and *OsGSTU51*/*U52 *showed divergent expression patterns (Fig. 5, Additional file 7). In fact, one of the members in these gene pairs is expressed at very low level in all the tissues/organs examined suggesting their non/hypo-functionalization. Among the six clusters of tandemly duplicated GST genes, the two genes included in each of cluster 2 (*OsGSTU39*/*U40*) and 6 (*OsGSTZ1*/*Z2*), exhibited highly similar expression patterns with some quantitative differences (Additional file 7). The GST genes included in other four clusters exhibited similar to highly diverged expression patterns indicating the events of neo-, sub-, and non/hypo-functionalization (Fig. 5, Additional file 7). The results suggest the evolution of GST gene family has occurred by gene duplication followed by retention due to sub- or neo-functionalization of the duplicated genes.

To investigate the probable explanation for divergence in expression patterns of duplicated GST genes, we analyzed their promoter sequences 1 kb upstream of translational start site. The putative cis-regulatory elements were identified using PLACE (a database of plant cis-acting regulatory DNA elements) search. This analysis revealed that the regulatory elements are more conserved in duplicated GST genes with similar expression patterns (for example, *OsEF1G1*/*EF1G2 *and *OsGSTF6*/*F14*) as compared to the GST genes with divergent expression patterns (for example, *OsGSTF12*/*F17 *and *OsGSTU51*/*U52*). The considerable difference in the regulatory elements of duplicated genes might explain their divergent expression patterns. However, experimental validation is required to reach this conclusion. Further, the existence of some other regulatory mechanism, which is responsible for divergent expression patterns and/or non-functionalization of one of the duplicates, can not be ruled out.

### Differential expression of GST genes during hormone treatment

Some of the plant GSTs are induced by plant hormones auxins and cytokinins. The transcript level of GST genes is induced very rapidly in the presence of auxin [37,38]. In this study, we used two microarray datasets to assess the effect of auxin and cytokinin on the expression profiles of GST genes. First dataset includes microarray analysis of 7-day-old rice seedlings treated with indole-3-acetic acid (IAA) and benzyl aminopurine (BAP) up to 3 h each [38]. Second dataset includes microarray analysis of root and leaf tissues of two-week-old seedlings treated with trans-zeatin (tZ) for 30 min and 120 min [39]. The data analysis showed that a total of 31 GST genes exhibit significant differential expression under at least one of the conditions analyzed (Additional file 8, 9). Interestingly, majority (27) of them belonged to Tau class. Quite a large number (14) of GST genes showed differential expression in the presence of auxin. All but one (*OsGSTF10*) of these 14 genes were up-regulated significantly. A total of 24 genes were differentially expressed in the presence of cytokinin. Three genes which showed up-regulation in the presence of BAP in first dataset did not show any differential expression in the presence of tZ in second dataset. Among the 21 genes which showed differential expression in the second dataset, 11 genes were differentially expressed in roots as compared to 15 in leaf. Five genes showed differential expression both in roots and leaf, whereas six and ten genes were unique to root and leaf, respectively. These results suggest the differential response of rice GST genes with respect to age of seedlings, tissue-type and/or cytokinin type. The differential expression of some representative genes in the presence of IAA and/or BAP has also been validated by real-time PCR analysis (Additional file 8).

### Differential expression of GST genes during abiotic stress

**Figure 6:**
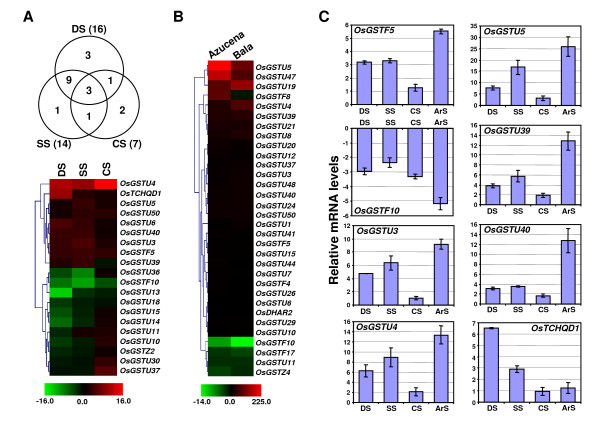
**Differential expression of rice GST genes in response to various abiotic and arsenate stress conditions**. Hierarchical clustering of GST genes showing significant differential expression under at least one abiotic stress condition **(A) **or arsenate stress **(B) **is shown. The fold change values (Additional files 10, 11) in treated sample as compared to its corresponding mock-treated control sample were used for clustering. The color scale for fold change values is shown at the bottom. Venn diagram represents number of GST genes commonly or specifically regulated by various abiotic stress conditions. **(C) **Real-time PCR analysis of selected genes to validate their differential expression during various abiotic stress conditions. The mRNA levels for each gene in different tissue samples were calculated relative to its expression in control seedlings. The error bars represent standard deviation. DS, desiccation stress; SS, salt stress; CS, cold stress; ArS, arsenate stress. Azucena and Bala represent arsenate-sensitive and arsenate-resistant rice varieties, respectively.

Many GSTs have been implicated in various abiotic stress responses in plants. The differential expression of several *Arabidopsis *GSTs in response to ethylene, jasmonic acid, salicylic acid, hydrogen peroxide and 2, 4-dichlorophenoxy acetic acid have been reported [6,40]. To study the effect of various abiotic stresses (desiccation, salt, cold and arsenate) on the expression profiles of GST genes, microarray data available under series accession number GSE6901 [30] was analyzed. Differential expression analysis for rice seedlings treated with different abiotic stresses (desiccation, salt and cold) as compared to mock-treated control seedlings was performed. This analysis showed that at least 20 GST genes (16 of Tau class, two of Phi class and one each of Zeta and TCHQD class) were differentially expressed significantly under at least one of the abiotic stress conditions analyzed. Among these, three GST genes were differentially expressed in all the stress conditions (Fig. 6A, Additional file 10). Other 11 genes were differentially expressed in any two stress conditions. However, six genes showed differential expression under only specific stress condition. One gene (*OsGSTU10*) which was down-regulated by desiccation stress was up-regulated by cold stress. Similarly, we analyzed the microarray data for arsenate stress from an earlier study [41]. The data included expression analysis from rice varieties Azucena (arsenate-sensitive) and Bala (arsenate-tolerant) grown in the presence or absence of 13.3 μm sodium arsenate for 7 days. The data analysis showed that a large number (32) of GST genes (25 of Tau class, five of Phi class and one each of Zeta and DHAR class) showed significant differential expression in response to arsenate stress as compared to other abiotic stress (20) (Fig. 6B, Additional file 11). A total of 27 GST genes were significantly up-regulated, whereas four genes were down-regulated in Azucena. In Bala, 23 genes were up-regulated and four genes were down-regulated. Twenty two GST genes were significantly up-regulated and four were down-regulated both in Azucena and Bala. Furthermore, 13 GST genes were commonly differentially expressed under various abiotic and arsenate stress conditions analyzed in this study. The real-time PCR analysis confirmed the differential expression of representative GST genes during various abiotic stress conditions and arsenate stress (Fig. 6C).

### Differential expression of GST genes during biotic stress

**Figure 7:**
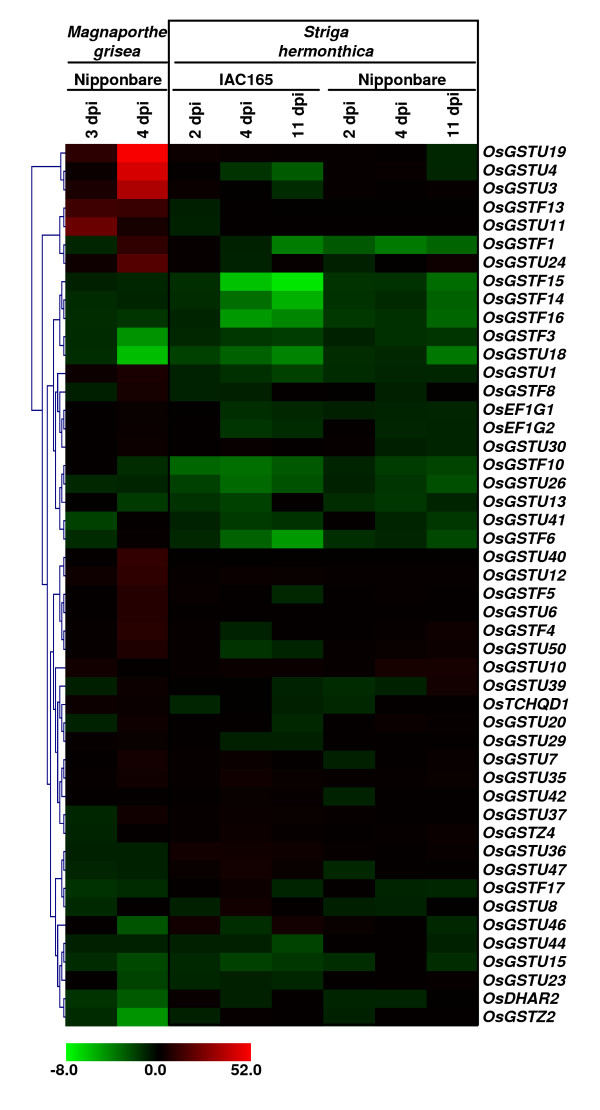
**Differential expression of rice GST genes in response to various biotic stress conditions**. Hierarchical clustering of GST genes showing significant differential expression in at least one condition is shown. The fold change values (Additional file 12) in treated sample as compared to its corresponding mock-treated control sample were used for clustering. The color scale for fold change values is shown at the bottom. Dpi, days post-inoculation.

GSTs have been shown to be differentially regulated upon pathogen attack in several plant species [6,42]. One of the most serious and widespread diseases of rice is blast caused by the ascomycete fungus *Magnaporthe grisea*. Recently, a transcriptome analysis of a fully susceptible infection of rice (cultivar Nipponbare) by a compatible *M. grisea *isolate (FR13) was performed to understand the molecular mechanism involved in their interaction [43]. We analyzed this data to have inkling about the role of rice GST genes in this host-pathogen interaction. The data includes microarray analysis of two-week-old rice seedlings (cultivar Nipponbare) treated with *M. grisea *(virulent isolate FR13) spore suspension on gelatine or gelatine alone after 3 days (3 dpi, without disease symptoms) and 4 days (4 dpi, with disease symptoms) post inoculation. The data analysis revealed that quite a large number (34) of GST genes are differentially expressed more than 2-fold (Fig. 7, Additional file 12). Among these, only 11 genes showed differential expression at 3 dpi, whereas 32 genes at 4 dpi (nine genes showed differential expression at both 3 and 4 dpi).

*Striga hermonthica *is an obligate root hemiparasite of rice and other cereals that causes severe loss of yield. To understand the possible interaction between rice roots and parasitic plant *S. hermonthica *at molecular level, global gene expression profiling was performed by Swarbrick and colleagues [44]. We took advantage of the availability of this data to study the expression profiles of rice GST genes in roots of susceptible (IAC165) and highly resistant (Nipponbare) cultivars in response to infection with *S. hermonthica *after 2, 4 and 11 dpi. The data analysis revealed that at least 17 genes were significantly up- and down-regulated by more than 2-fold in Nipponbare as compared with 28 genes in susceptible IAC165 cultivar (Fig. 7, Additional file 12). In IAC165, 15 and 13 genes were up- and down-regulated, respectively, whereas nine and eight GST genes were up- and down-regulated, respectively, in Nipponbare. Twelve GST genes showed differential expression in both cultivars. Among the total 48 GST genes differentially expressed in two datasets, 30 genes belonged to Tau class, 12 to Phi, two to Zeta, two to EF1G and one each to DHAR and TCHQD classes.

### Overlap of GST responses to various stimuli and developmental processes

**Figure 8:**
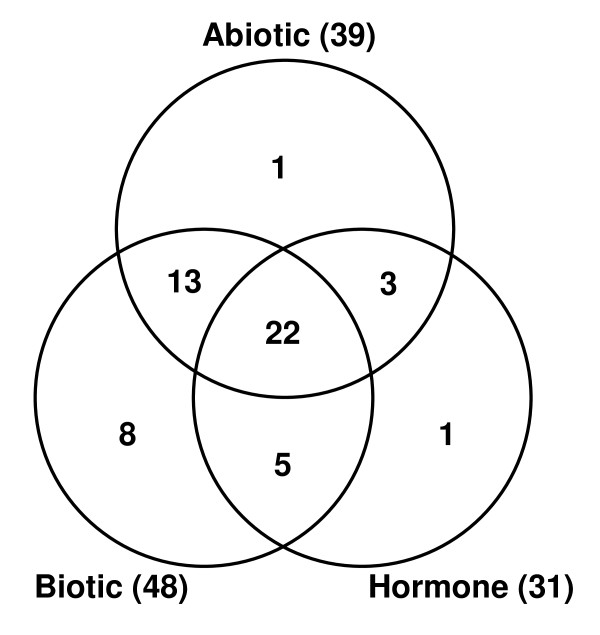
**Venn diagram representing the number of GST genes commonly or specifically regulated by various environmental stimuli, including plant hormones, abiotic stress and/or biotic stress**.

The overlap of response of GST genes to various stimuli, including hormone, abiotic stress (including arsenate stress) and biotic stress, and developmental processes was analyzed. A global figure including the expression profiles of all the 71 GST genes for which microarray data was available, during various stages of development and under various environmental stimuli analyzed in this study, was generated (Additional file 13). It is noteworthy that among a total of 53 GST genes, which were differentially expressed in the presence of any of these three environmental stimuli (hormone, abiotic stress and biotic stress), 22 genes showed response to all of these stimuli (Fig. 8). Other 21 GST genes responded to any of the two stimuli. However, 15 GST genes showed response to specific stimuli (Fig. 8). Some of the GST family members were induced by multiple stresses, while others showed response to few to unique stress condition(s). For example, *OsGSTF5*, *U3*, *U4*, *U6*, *U37*, *U39*, *U40 *and *U50 *were induced by abiotic, arsenate and biotic stresses analyzed in this study. However, *OsGSTU18*, *U36*, *TCHQD1 *exhibited response to abiotic and biotic stresses, but were non-responsive to arsenate stress. Likewise, *OsGSTU14 *responded specifically to abiotic stress, *OsGSTU21 *and *U48 *to arsenate stress and *OsGSTF1*, *F13 *and *U35 *to biotic stress. In general, the members of Tau class appeared to show response to various stimuli more frequently. However, 71% (12) of Phi class GST genes responded to biotic stress as compared to 58% (30) of Tau class. The expression of two GST genes of Tau class, *OsGSTU3 *and *U4*, was found to be induced by heavy metals, hypoxic stress and salt stress [45]. We also found that the transcript levels of these genes are induced by desiccation, salt and cold stress. In addition, our analysis revealed that the transcript levels of these genes are induced in the presence of arsenate and biotic stress as well, indicating their role in broad spectrum stress responses.

Given that plant GSTs are induced by a plethora of environmental factors, it was proposed that GST expression is universally induced by the production of stress-associated active oxygen species (AOS) signaling molecules, which in turn counteracts the adverse effects of AOS by their GSH-dependent peroxidase activity [12]. However, our study showed that, although several GSTs are commonly regulated, many of them exhibit differential and specific response to various stresses as well. Similar observations have been reported in other studies too [6,15]. These results indicate that the induction of GST family members occurs via multiple and independent pathways and a subset of which may involve AOS as signaling molecules. It has been reported that GSTs are induced very rapidly by pathogens and typically precede the induction of well-known defense genes such as pathogenesis-related proteins [42,46,47]. Based on analysis of mutants, this rapid induction of GSTs has been found to be dependent on combined SA- and ethylene signaling [47]. This was further supported by the increased production of SA and ethylene by the plants inoculated with pathogen [47]. This clearly indicates a cross-talk between various signaling pathways.

Many evidences show crosstalk between various developmental processes and environmental stimuli [30,38,48]. We also found relation between tissue-/developmental stage-specific expression pattern and stress responses of few GST genes. For example, *OsGSTU4 *is preferentially expressed in the root and stages of seed development and was also found to be highly up-regulated by plant hormones, abiotic stress, arsenate stress and biotic stress conditions. Likewise, *OsGSTU5 *and *OsGSTU37 *are preferentially expressed in root and are also up-regulated by auxin and various stress conditions. The *OsGSTF10 *was specifically expressed in root and was down-regulated by most of the environmental stimuli analyzed in this study. Other GST genes, *OsGSTU10*, *U18 *and *U36*, which were preferentially expressed in root, were also down-regulated by one or more of the stimuli. These commonly regulated GST genes might mediate plant growth responses to various environmental stimuli in specific tissues/organs and/or developmental stage.

## Conclusion

This study provides not only an updated annotation and nomenclature of the GST family in rice, but also the identification of several tissue- and/or developmental stage-specific, hormone-responsive and abiotic and biotic stress-responsive GST genes included in various classes. Considering the fact that a very limited number of GST genes have been characterized till date, our results provide a very useful framework and starting point for revealing the function(s) of GST family members in rice, especially those involved in specific developmental processes, hormone response and stress tolerance.

## Methods

### Database search and sequence analysis

GSTs were identified by keyword, domain name and HMMER searches of rice proteome available at Rice Genome Annotation Project [49] database using the Hidden Markov Model (HMM) profile (build 2.3.2) of GST_N domain (PF02798) downloaded from PFam. The presence of GST_N domain in individual protein was further confirmed by SMART analysis. Multiple sequence alignment analyses were performed using ClustalX (version 1.83) program. The GST genes present on duplicated chromosomal segments were identified by segmental genome duplication of rice available at RGAP with the maximum length distance permitted between collinear gene pairs of 500 kb. The GST genes separated by a maximum of five genes were identified as tandemly duplicated genes. The unrooted phylogenetic trees were constructed by neighbor-joining method and displayed using Treeview program. Putative conserved motifs were identified using MEME (version 4.1.0) program [50].

### Plant growth

The tissue samples of mature leaf, Y leaf and various stages of panicle and seed development were collected from field grown rice (*Oryza sativa *ssp. *indica *var. IR64) plants as described [30]. Roots were harvested from 7-day-old seedlings grown hydroponically. For salt, desiccation, cold and arsenate stress treatments, 7-day-old light-grown rice (*Oryza sativa *L. ssp. *indica *var. IR64) seedlings were transferred to a beaker containing 200 mM NaCl solution, dried between folds of tissue paper at 28 ± 1°C, kept at 4 ± 1°C and, transferred to a beaker containing 50 μM sodium arsenate solution, respectively, each for 3 h. Likewise, 7-day-old light-grown rice seedlings were transferred to a beaker containing 50 μM solution of indole-3-acetic acid and 50 μM solution of benzyl aminopurine for auxin and cytokinin treatment, respectively. The control seedlings were kept in water for 3 h, at 28 ± 1°C.

### Microarray data analysis

The microarray data publicly available at GEO database under the series accession numbers GSE6893 (expression data for reproductive development), GSE7951 (expression profiling of stigma), GSE6901 (expression data for stress treatment), GSE4471 (expression data from rice varieties Azucena and Bala grown in arsenate), GSE5167 (expression data for auxin and cytokinin response), GSE6719 (expression data for cytokinin response), GSE7256 (expression data for virulent infection by *Magnaporthe grisea*), and GSE10373 (expression data for interaction with the parasitic plant *Striga hermonthica*) were used for expression analysis of rice GST genes. The entire microarray experiments used in this study are listed in Additional file 5. The Affymetrix CEL files were imported into Genespring GX (version 10) software (Agilent Technologies). The normalization and probe summarization was performed by Gene Chip Robust Multi Array (GCRMA) method. We performed a stringent statistical analysis consisting of one-way ANOVA over all the samples in a series and the Benjamini-Hoschberg multiple testing correction was applied to the data (P ≤ 0.05).

The IDs of probe sets present on the Affymetrix rice genome array representing the GST genes were identified using Rice Multi-platform Microarray Search [51] tool. The data for only one probe set for each GST gene was used for expression analysis. This resulted in identification of probe sets for 71 GST genes that were represented on the Affymetrix rice genome array. After normalization and log transformation of data for all the rice genes present on the chip, the log signal intensity values for rice probe IDs corresponding to GST genes were extracted as a subset and all the subsequent analyses were done on this subset only. The genes that are up- or down-regulated equal to or more than two-fold with a *P*-value of at least 0.05 were considered to be differentially expressed significantly. We generated tab-delimited files for average log signal values for development data and fold-change values for abiotic stress, biotic stress and hormone treatments and imported them into TIGR MultiExperiment Viewer (MeV) [52] to carry out clustering analysis. Hierarchical clustering was performed based on Euclidean distance matrix and Complete Linkage rule.

### Real-time PCR analysis

To confirm the differential expression of representative GST genes in various rice tissues/developmental stages and stress/hormone treatments identified by microarray data analysis, real-time PCR analysis was performed using gene-specific primers as described earlier [53]. The primer sequences are listed in Additional file 14. At least three biological replicates of each sample and three technical replicates of each biological replicate were analyzed for real-time PCR analysis. The expression of each gene in different RNA samples was normalized with the expression of the suitable internal control gene, *UBQ5 *[53] to ensure the equal amount of cDNA used for individual reactions. The mRNA levels for each candidate gene in different tissue samples were calculated using the ΔΔC_T _method.

### MPSS data analysis

Expression evidence from MPSS (Massively Parallel Signature Sequencing) tags was determined from the Rice MPSS project [32,54]. The signature was considered to be significant if it uniquely identifies an individual gene and shows perfect match (100% identity over 100% of the length of the tag). The normalized abundance (tags per million, tpm) of these signatures for a given gene in a given library represents the quantitative estimate of expression of that gene. MPSS data for 17-base signatures from 22 mRNA libraries representing 18 different tissues/organs of rice (Additional file 4) were used for the analysis.

## Authors' contributions

MJ conceived the study, carried out data analysis and wrote the manuscript. CG participated in data analysis at initial stage and real-time PCR experiments. AB participated in real-time PCR experiments and promoter analysis of duplicated genes. All authors read and approved the final manuscript.

## Supplementary Material

Additional file 1 **GST gene family in rice**. The gene name, locus ID, open reading frame length, protein length and chromosomal location of all the 79 GST genes are given. Click here for file

Additional file 2 **Phylogenetic relationship and classification of rice GST genes**. The unrooted tree was constructed based on multiple sequence alignment of full-length protein sequences using ClustalX program by neighbor-joining method with 1000 bootstrap replicates. The open circles and filled rectangles represent bootstrap values of 50-80% and >80%, respectively. The bar indicates 0.1 substitutions per site. All the rice GST proteins grouped into seven classes. Click here for file

Additional file 3 **Organization of 10 motifs predicted by MEME in rice GST proteins**. The position of motifs predicted in the GST proteins has been shown. The numbers 1 - 10 in boxes indicate the motifs 1 - 10 given in Fig. 2. Click here for file

Additional file 4 **MPSS data showing tissue-specific abundance of rice GST genes**. The number of tags (in tpm) for each GST gene in different tissue samples is given. Click here for file

Additional file 5 Summary of rice microarray experiments from GEO database used in this study. Click here for file

Additional file 6 Average log signal values of 71 GST genes in various tissues/organs and developmental stages. Click here for file

Additional file 7 **Expression patterns of duplicated GST genes**. The average log signal values (from three biological replicates) for each gene in all the samples analyzed is presented on Y-axis. Click here for file

Additional file 8 **Differential expression of rice GST genes in response to plant hormones auxin and cytokinin**. **(A) **Hierarchical clustering of GST genes showing significant differential expression in at least one condition is shown. The fold change values in treated sample as compared to its corresponding mock-treated control sample were used for clustering. The color scale for fold change values is shown at the bottom. IAA, indole-3-acetic acid treatment; BAP, benzyl aminopurine treatment; tZ, trans-zeatin treatment. **(B) **Real-time PCR analysis of representative GST genes to validate their differential expression during auxin (IAA) and cytokinin (BAP) treatment. The mRNA levels for each gene in different tissue samples were calculated relative to its expression in control seedlings. The error bars represent standard deviation. Click here for file

Additional file 9 Fold change and regulation of GST genes differentially expressed in the presence of plant hormones auxin and cytokinin. Click here for file

Additional file 10 Fold change and regulation of GST genes differentially expressed under various abiotic stress conditions. Click here for file

Additional file 11 Fold change and regulation of GST genes differentially expressed under arsenate stress. Click here for file

Additional file 12 Fold change and regulation of GST genes differentially expressed under various biotic stress conditions. Click here for file

Additional file 13 **Expression patterns of rice GST genes in various tissues/organs/developmental stages and environmental conditions (various hormone, abiotic stress, arsenate stress and abiotic stress treatments)**. Hierarchical clustering analysis of 71 GST genes represented on Affymetrix Rice Genome Array is shown. For clustering we used average log signal values for two/three biological replicates of each sample after normalization of the raw data. The color scale for log signal values is shown at the bottom. Click here for file

Additional file 14 Primer sequences used for real-time PCR analysis. Click here for file
