# Supplementary material for: Comprehensive expression analysis suggests overlapping and specific roles of rice glutathione S-transferase genes during development and stress responses
Source: BMC Genomics. 2010 Jan 29;11:73. doi: 10.1186/1471-2164-11-73 (PMC2825235; doi:10.1186/1471-2164-11-73)

Additional file 2 Phylogenetic relationship and classification of rice GST genes. The unrooted tree was constructed based on multiple sequence alignment of full-length protein sequences using ClustalX program by neighbor-joining method with 1000 bootstrap replicates. The open circles and filled rectangles represent bootstrap values of 50-80% and >80%, respectively. The bar indicates 0.1 substitutions per site. All the rice GST proteins grouped into seven classes.

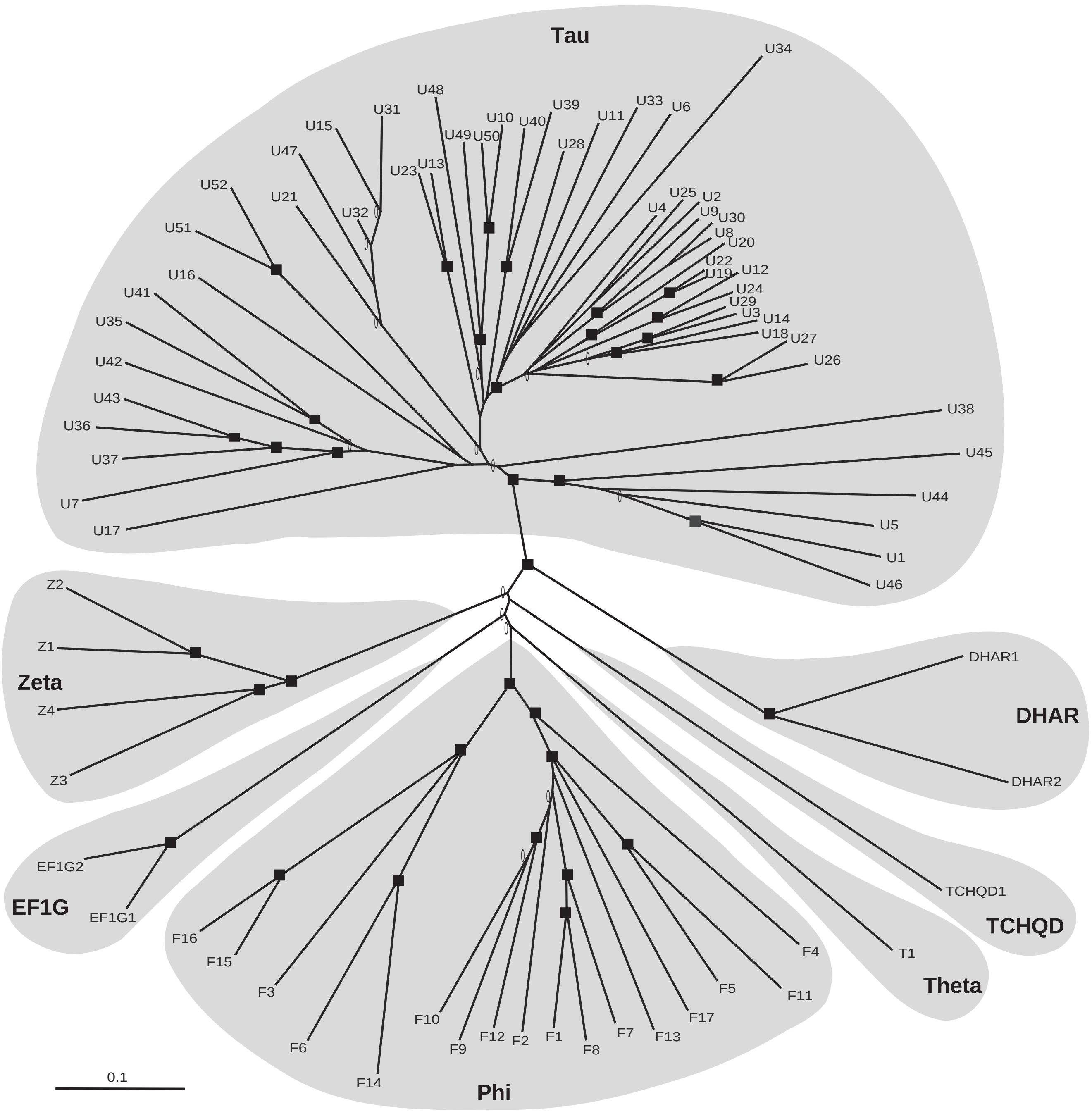

Supplement: Additional file 2 — Phylogenetic relationship and classification of rice GST genes. The unrooted tree was constructed based on multiple sequence alignment of full-length protein sequences using ClustalX program by neighbor-joining method with 1000 bootstrap replicates. The open circles and filled rectangles represent bootstrap values of 50-80% and >80%, respectively. The bar indicates 0.1 substitutions per site. All the rice GST proteins grouped into seven classes. [file 1471-2164-11-73-S2.PDF]
