# Supplementary material for: Comprehensive expression analysis suggests overlapping and specific roles of rice glutathione S-transferase genes during development and stress responses
Source: BMC Genomics. 2010 Jan 29;11:73. doi: 10.1186/1471-2164-11-73 (PMC2825235; doi:10.1186/1471-2164-11-73)
Supplement: Additional file 3 — Organization of 10 motifs predicted by MEME in rice GST proteins. The position of motifs predicted in the GST proteins has been shown. The numbers 1 - 10 in boxes indicate the motifs 1 - 10 given in Fig. 2. [file 1471-2164-11-73-S3.DOC]

**Additional file 3.** Organization of 10 motifs predicted by MEME in rice GST proteins. The numbers 1 – 10 in boxes indicate the motifs 1 – 10 given in Figure 2.

| **Name** | **Motifs** |
| --- | --- |
| OsGSTU19 | |  | 5 |  | 1 | 4 |  | 2 |  | 8 |  | 3 |  | 6 |  |  | | --- | --- | --- | --- | --- | --- | --- | --- | --- | --- | --- | --- | --- | --- | --- | |
| OsGSTU22 | |  | 5 |  | 1 | 4 |  | 2 |  | 8 |  | 3 |  | 6 |  |  | | --- | --- | --- | --- | --- | --- | --- | --- | --- | --- | --- | --- | --- | --- | --- | |
| OsGSTU29 | |  | 5 |  | 1 | 4 |  | 2 |  | 8 |  | 3 |  | 6 |  |  | | --- | --- | --- | --- | --- | --- | --- | --- | --- | --- | --- | --- | --- | --- | --- | |
| OsGSTU18 | |  | 5 |  | 1 | 4 |  | 2 |  | 8 |  | 3 |  | 6 |  |  | | --- | --- | --- | --- | --- | --- | --- | --- | --- | --- | --- | --- | --- | --- | --- | |
| OsGSTU14 | |  | 5 |  | 1 | 4 |  | 2 |  | 8 |  | 3 |  | 6 |  |  | | --- | --- | --- | --- | --- | --- | --- | --- | --- | --- | --- | --- | --- | --- | --- | |
| OsGSTU8 | |  | 5 |  | 1 | 4 |  | 2 |  | 8 |  | 3 |  | 6 |  |  | | --- | --- | --- | --- | --- | --- | --- | --- | --- | --- | --- | --- | --- | --- | --- | |
| OsGSTU3 | |  | 5 |  | 1 | 4 |  | 2 |  | 8 |  | 3 |  | 6 |  |  | | --- | --- | --- | --- | --- | --- | --- | --- | --- | --- | --- | --- | --- | --- | --- | |
| OsGSTU24 | |  | 5 |  | 1 | 4 |  | 2 |  | 8 |  | 3 |  | 6 |  |  | | --- | --- | --- | --- | --- | --- | --- | --- | --- | --- | --- | --- | --- | --- | --- | |
| OsGSTU9 | |  | 5 |  | 1 | 4 |  | 2 |  | 8 |  | 3 |  | 6 |  |  | | --- | --- | --- | --- | --- | --- | --- | --- | --- | --- | --- | --- | --- | --- | --- | |
| OsGSTU30 | |  | 5 |  | 1 | 4 |  | 2 |  | 8 |  | 3 |  | 6 |  |  | | --- | --- | --- | --- | --- | --- | --- | --- | --- | --- | --- | --- | --- | --- | --- | |
| OsGSTU25 | |  | 5 |  | 1 | 4 |  | 2 |  | 8 |  | 3 |  | 6 |  |  | | --- | --- | --- | --- | --- | --- | --- | --- | --- | --- | --- | --- | --- | --- | --- | |
| OsGSTU4 | |  | 5 |  | 1 | 4 |  | 2 |  | 8 |  | 3 |  | 6 |  |  | | --- | --- | --- | --- | --- | --- | --- | --- | --- | --- | --- | --- | --- | --- | --- | |
| OsGSTU12 | |  | 5 |  | 1 | 4 |  | 2 |  | 8 |  | 3 |  | 6 |  |  | | --- | --- | --- | --- | --- | --- | --- | --- | --- | --- | --- | --- | --- | --- | --- | |
| OsGSTU13 | |  | 5 |  | 1 | 4 |  | 2 |  | 8 |  | 3 |  | 6 |  |  | | --- | --- | --- | --- | --- | --- | --- | --- | --- | --- | --- | --- | --- | --- | --- | |
| OsGSTU50 | |  | 5 |  | 1 | 4 |  | 2 |  | 8 |  | 3 |  | 6 |  |  | | --- | --- | --- | --- | --- | --- | --- | --- | --- | --- | --- | --- | --- | --- | --- | |
| OsGSTU23 | |  | 5 |  | 1 | 4 |  | 2 |  | 8 |  | 3 |  | 6 |  |  | | --- | --- | --- | --- | --- | --- | --- | --- | --- | --- | --- | --- | --- | --- | --- | |
| OsGSTU20 | |  | 5 |  | 1 | 4 |  | 2 |  | 8 |  | 3 |  | 6 |  |  | | --- | --- | --- | --- | --- | --- | --- | --- | --- | --- | --- | --- | --- | --- | --- | |
| OsGSTU27 | |  | 5 |  | 1 | 4 |  | 2 |  | 8 |  | 3 |  | 6 |  |  | | --- | --- | --- | --- | --- | --- | --- | --- | --- | --- | --- | --- | --- | --- | --- | |
| OsGSTU2 | |  | 5 |  | 1 | 4 |  | 2 |  | 8 |  | 3 |  | 6 |  |  | | --- | --- | --- | --- | --- | --- | --- | --- | --- | --- | --- | --- | --- | --- | --- | |
| OsGSTU26 | |  | 5 |  | 1 | 4 |  | 2 |  | 8 |  | 3 |  | 6 |  |  | | --- | --- | --- | --- | --- | --- | --- | --- | --- | --- | --- | --- | --- | --- | --- | |
| OsGSTU49 | |  | 5 |  | 1 | 4 |  | 2 |  | 8 |  | 3 |  | 6 |  |  | | --- | --- | --- | --- | --- | --- | --- | --- | --- | --- | --- | --- | --- | --- | --- | |
| OsGSTU10 | |  | 5 |  | 1 | 4 |  | 2 |  | 8 |  | 3 |  | 6 |  |  | | --- | --- | --- | --- | --- | --- | --- | --- | --- | --- | --- | --- | --- | --- | --- | |
| OsGSTU28 | |  | 5 |  | 1 | 4 |  | 2 |  | 3 |  | 6 |  |  | | --- | --- | --- | --- | --- | --- | --- | --- | --- | --- | --- | --- | --- | |
| OsGSTU40 | |  | 5 |  | 1 | 4 |  | 2 |  | 8 |  | 3 |  | 6 |  |  | | --- | --- | --- | --- | --- | --- | --- | --- | --- | --- | --- | --- | --- | --- | --- | |
| OsGSTU39 | |  | 5 |  | 1 | 4 |  | 2 |  | 8 |  | 3 |  | 6 |  |  | | --- | --- | --- | --- | --- | --- | --- | --- | --- | --- | --- | --- | --- | --- | --- | |
| OsGSTU48 | |  | 5 |  | 1 | 4 |  | 2 |  | 8 |  | 3 |  | 6 |  |  | | --- | --- | --- | --- | --- | --- | --- | --- | --- | --- | --- | --- | --- | --- | --- | |
| OsGSTU33 | |  | 5 |  | 1 | 4 |  | 2 |  | 8 |  | 3 |  | 6 |  |  | | --- | --- | --- | --- | --- | --- | --- | --- | --- | --- | --- | --- | --- | --- | --- | |
| OsGSTU43 | |  | 5 |  | 1 | 4 |  | 2 |  | 3 |  | 6 |  |  | | --- | --- | --- | --- | --- | --- | --- | --- | --- | --- | --- | --- | --- | |
| OsGSTU11 | |  | 5 |  | 1 | 4 |  | 2 |  | 8 |  | 3 |  | 6 |  |  | | --- | --- | --- | --- | --- | --- | --- | --- | --- | --- | --- | --- | --- | --- | --- | |
| OsGSTU37 | |  | 5 |  | 1 | 4 |  | 2 |  | 3 |  | 6 |  |  | | --- | --- | --- | --- | --- | --- | --- | --- | --- | --- | --- | --- | --- | |
| OsGSTU15 | |  | 5 |  | 1 | 4 |  | 2 |  | 8 |  | 3 |  | 6 |  |  | | --- | --- | --- | --- | --- | --- | --- | --- | --- | --- | --- | --- | --- | --- | --- | |
| OsGSTU6 | |  | 5 |  | 1 | 4 |  | 2 |  | 8 |  | 3 |  | 6 |  |  | | --- | --- | --- | --- | --- | --- | --- | --- | --- | --- | --- | --- | --- | --- | --- | |
| OsGSTU36 | |  | 5 |  | 1 | 4 |  | 2 |  | 3 |  | 6 |  |  | | --- | --- | --- | --- | --- | --- | --- | --- | --- | --- | --- | --- | --- | |
| OsGSTU47 | |  | 1 | 4 |  | 2 |  | 8 |  | 3 |  | 6 |  |  | | --- | --- | --- | --- | --- | --- | --- | --- | --- | --- | --- | --- | --- | |
| OsGSTU35 | |  | 5 |  | 1 | 4 |  | 2 |  | 3 |  | 6 |  |  | | --- | --- | --- | --- | --- | --- | --- | --- | --- | --- | --- | --- | --- | |
| OsGSTU17 | |  | 5 |  | 1 | 4 |  | 2 |  | 3 |  | 6 |  |  | | --- | --- | --- | --- | --- | --- | --- | --- | --- | --- | --- | --- | --- | |
| OsGSTU5 | |  | 5 |  | 1 | 4 |  | 2 |  | 3 |  | 6 |  |  | | --- | --- | --- | --- | --- | --- | --- | --- | --- | --- | --- | --- | --- | |
| OsGSTU42 | |  | 5 |  | 1 |  | 4 |  | 2 |  | 3 |  | 6 |  |  | | --- | --- | --- | --- | --- | --- | --- | --- | --- | --- | --- | --- | --- | --- | |
| OsGSTU16 | |  | 5 |  | 1 | 4 |  | 2 |  | 8 |  | 3 |  | 6 |  |  | | --- | --- | --- | --- | --- | --- | --- | --- | --- | --- | --- | --- | --- | --- | --- | |
| OsGSTF1 | |  | 5 |  | 7 |  | 9 | 10 |  |  | | --- | --- | --- | --- | --- | --- | --- | --- | --- | |
| OsGSTF7 | |  | 5 |  | 7 |  | 9 | 10 |  |  | | --- | --- | --- | --- | --- | --- | --- | --- | --- | |
| OsGSTU41 | |  | 5 |  | 1 |  | 4 |  | 2 |  | 8 |  | 3 |  | 6 |  |  | | --- | --- | --- | --- | --- | --- | --- | --- | --- | --- | --- | --- | --- | --- | --- | --- | |
| OsGSTF8 | |  | 5 |  | 7 |  | 9 | 10 |  |  | | --- | --- | --- | --- | --- | --- | --- | --- | --- | |
| OsGSTF5 | |  | 5 |  | 7 |  | 9 | 10 |  |  | | --- | --- | --- | --- | --- | --- | --- | --- | --- | |
| OsGSTF10 | |  | 5 |  | 7 |  | 9 | 10 |  |  | | --- | --- | --- | --- | --- | --- | --- | --- | --- | |
| OsGSTU31 | |  | 5 |  | 1 | 4 |  | 8 |  | 3 |  | 6 |  |  | | --- | --- | --- | --- | --- | --- | --- | --- | --- | --- | --- | --- | --- | |
| OsGSTF12 | |  | 5 |  | 7 |  | 9 | 10 |  |  | | --- | --- | --- | --- | --- | --- | --- | --- | --- | |
| OsGSTF2 | |  | 5 |  | 7 |  | 9 | 10 |  |  | | --- | --- | --- | --- | --- | --- | --- | --- | --- | |
| OsGSTU1 | |  | 5 |  | 1 | 4 |  | 2 |  | 8 |  | 3 |  | 6 |  |  | | --- | --- | --- | --- | --- | --- | --- | --- | --- | --- | --- | --- | --- | --- | --- | |
| OsGSTU7 | |  | 5 |  | 1 |  | 4 |  | 2 |  | 3 |  | 6 |  |  | | --- | --- | --- | --- | --- | --- | --- | --- | --- | --- | --- | --- | --- | --- | |
| OsGSTU52 | |  | 1 | 4 |  | 2 |  | 3 |  | 6 |  |  | | --- | --- | --- | --- | --- | --- | --- | --- | --- | --- | --- | |
| OsGSTU34 | |  | 5 |  | 1 |  | 4 |  | 2 |  | 8 |  | 3 |  | 6 |  |  | | --- | --- | --- | --- | --- | --- | --- | --- | --- | --- | --- | --- | --- | --- | --- | --- | |
| OsGSTF9 | |  | 5 |  | 7 |  | 9 | 10 |  |  | | --- | --- | --- | --- | --- | --- | --- | --- | --- | |
| OsGSTF4 | |  | 5 |  | 7 |  | 9 | 10 |  |  | | --- | --- | --- | --- | --- | --- | --- | --- | --- | |
| OsGSTU44 | |  | 5 |  | 1 | 4 |  | 2 |  | 8 |  | 3 |  | 6 |  |  | | --- | --- | --- | --- | --- | --- | --- | --- | --- | --- | --- | --- | --- | --- | --- | |
| OsGSTF3 | |  | 5 |  | 7 |  | 9 |  | 10 |  |  | | --- | --- | --- | --- | --- | --- | --- | --- | --- | --- | |
| OsGSTF16 | |  | 5 |  | 7 |  | 2 |  | 9 |  | 10 |  |  | | --- | --- | --- | --- | --- | --- | --- | --- | --- | --- | --- | --- | |
| OsGSTU46 | |  | 5 |  | 1 |  | 4 |  | 2 |  | 3 |  | 6 |  |  | | --- | --- | --- | --- | --- | --- | --- | --- | --- | --- | --- | --- | --- | --- | |
| OsGSTF15 | |  | 5 |  | 7 |  | 9 |  | 10 |  |  | | --- | --- | --- | --- | --- | --- | --- | --- | --- | --- | |
| OsGSTF6 | |  | 5 |  | 7 |  | 9 |  | 10 |  |  | | --- | --- | --- | --- | --- | --- | --- | --- | --- | --- | |
| OsGSTU21 | |  | 5 |  | 1 | 4 |  | 2 |  |  | | --- | --- | --- | --- | --- | --- | --- | --- | --- | |
| OsGSTF17 | |  | 5 |  | 7 |  | 9 |  |  | | --- | --- | --- | --- | --- | --- | --- | --- | |
| OsGSTF13 | |  | 5 |  | 7 |  | 9 |  | 10 |  |  | | --- | --- | --- | --- | --- | --- | --- | --- | --- | --- | |
| OsGSTU45 | |  | 1 | 4 |  | 2 |  | 8 |  | 6 |  |  | | --- | --- | --- | --- | --- | --- | --- | --- | --- | --- | --- | |
| OsGSTF11 | |  | 7 |  | 9 | 10 |  |  | | --- | --- | --- | --- | --- | --- | --- | |
| OsGSTF14 | |  | 5 |  | 7 |  | 9 |  | 10 |  |  | | --- | --- | --- | --- | --- | --- | --- | --- | --- | --- | |
| OsGSTU38 | |  | 5 |  | 4 |  | 2 |  | 8 |  | 3 |  |  | | --- | --- | --- | --- | --- | --- | --- | --- | --- | --- | --- | --- | |
| OsEF1G1 | |  | 7 |  | 9 |  | 10 |  |  | | --- | --- | --- | --- | --- | --- | --- | --- | |
| OsEF1G2 | |  | 7 |  | 9 |  | 10 |  |  | | --- | --- | --- | --- | --- | --- | --- | --- | |
| OsGSTZ1 | |  | 5 |  | 7 |  | 4 |  | 6 |  |  | | --- | --- | --- | --- | --- | --- | --- | --- | --- | --- | |
| OsGSTZ2 | |  | 5 |  | 7 |  | 6 |  |  | | --- | --- | --- | --- | --- | --- | --- | --- | |
| OsGSTZ3 | |  | 5 |  | 7 |  | 6 |  |  | | --- | --- | --- | --- | --- | --- | --- | --- | |
| OsGSTZ4 | |  | 5 |  | 7 |  | 6 |  |  | | --- | --- | --- | --- | --- | --- | --- | --- | |
| TCHQD1 | |  | 5 |  | 7 |  | 9 |  | 10 |  |  | | --- | --- | --- | --- | --- | --- | --- | --- | --- | --- | |
| OsDHAR2 | |  | 6 |  | 5 |  | 4 |  | 10 |  |  | | --- | --- | --- | --- | --- | --- | --- | --- | --- | --- | |
| OsDHAR1 | |  | 5 |  | 4 |  | 8 | 3 |  | 10 |  |  | | --- | --- | --- | --- | --- | --- | --- | --- | --- | --- | --- | |
| OsGSTU32 | |  | 5 |  | 1 |  |  | | --- | --- | --- | --- | --- | --- | |
| OsGSTU51 | |  | 5 |  | 1 |  |  | | --- | --- | --- | --- | --- | --- | |
| OsGSTT1 | |  | 7 |  | 2 |  |  | | --- | --- | --- | --- | --- | --- | |
