# Supplementary material for: Comprehensive expression analysis suggests overlapping and specific roles of rice glutathione S-transferase genes during development and stress responses
Source: BMC Genomics. 2010 Jan 29;11:73. doi: 10.1186/1471-2164-11-73 (PMC2825235; doi:10.1186/1471-2164-11-73)
Supplement: Additional file 5 — Summary of rice microarray experiments from GEO database used in this study. [file 1471-2164-11-73-S5.DOC]

**Additional file 5.** Summary of rice microarray experiments from GEO database used in this study.

| **S. no.** | **Series accession no.** | **Sample description** | **No. of replicates** | **Reference** |
| --- | --- | --- | --- | --- |
| **A) Development** | | | | |
| 1. | GSE6893 | Root, 7-day-old Seedling | 3 | Jain *et al*., 2007 |
|  |  | Mature leaf | 3 |  |
|  |  | Y Leaf | 3 |  |
|  |  | SAM (Shoot apical meristem) | 3 |  |
|  |  | Young inflorescence (P1, upto 3 cm) | 3 |  |
|  |  | Inflorescence (P2, 3 - 5 cm) | 3 |  |
|  |  | Inflorescence (P3, 5 - 10 cm) | 3 |  |
|  |  | Inflorescence (P4, 10 - 15 cm) | 3 |  |
|  |  | Inflorescence (P5, 15 - 22 cm) | 3 |  |
|  |  | Inflorescence (P6, 22 - 30 cm) | 3 |  |
|  |  | Seed (S1, 0 - 2 dap) | 3 |  |
|  |  | Seed (S2, 3 - 4 dap) | 3 |  |
|  |  | Seed (S3, 5 - 10 dap) | 3 |  |
|  |  | Seed (S4, 11 - 20 dap) | 3 |  |
|  |  | Seed (S5, 21 - 29 dap) | 3 |  |
| 2. | GSE7951 | Stigma | 3 | Li *et al*., 2007 |
|  |  | Ovary | 3 |  |
|  | | | | |
| **B) Abiotic stress** | | | | |
| 1. | GSE4471 | Rice Azucena 0ppm Arsenate (Control) | 3 | Norton *et al*., 2008 |
|  |  | Rice Azucena 1ppm Arsenate | 3 |  |
|  |  | Rice Bala 0ppm Arsenate (Control) | 3 |  |
|  |  | Rice Bala 1ppm Arsenate | 3 |  |
| 2. | GSE6901 | 7-day-old Seedling (control) | 3 | Jain *et al*., 2007 |
|  |  | Drought stress | 3 |  |
|  |  | Salt stress | 3 |  |
|  |  | Cold stress | 3 |  |
|  |  |  |  |  |
| **C) Biotic stress** | | | | |
| 1. | GSE7256 | mock 3 dpi | 2 | Ribot *et al*., 2008 |
|  |  | mock 4 dpi | 2 |  |
|  |  | FR13 3 dpi (Magnaporthe grisea) | 2 |  |
|  |  | FR13 4 dpi(Magnaporthe grisea) | 2 |  |
| 2. | GSE10373 | IAC165, 2 days after mock-infection | 2 | Swarbrick *et al*., 2008 |
|  |  | IAC165, 2 days after infection with S. hermonthica | 2 |  |
|  |  | IAC165, 4 days after mock-infection | 2 |  |
|  |  | IAC165, 4 days after infection with S. hermonthica | 2 |  |
|  |  | IAC165, 11 days after mock-infection | 2 |  |
|  |  | IAC165, 11 days after infection with S. hermonthica | 2 |  |
|  |  | Nipponbare, 2 days after mock-infection | 2 |  |
|  |  | Nipponbare, 2 days after infection with S. hermonthica | 2 |  |
|  |  | Nipponbare, 4 days after mock-infection | 2 |  |
|  |  | Nipponbare, 4 days after infection with S. hermonthica | 2 |  |
|  |  | Nipponbare, 11 days after mock-infection | 2 |  |
|  |  | Nipponbare, 11 days after infection with S. hermonthica | 2 |  |
|  |  |  |  |  |
| **D) Hormone** | | | | |
| 1. | GSE6719 | Root_DMSO-treatment_30min | 3 | Hirose *et al*., 2007 |
|  |  | Root_tZ-treatment_30min | 3 |  |
|  |  | Root_DMSO-treatment_120min | 3 |  |
|  |  | Root_tZ-treatment_120min | 3 |  |
|  |  | Leaf_DMSO-treatment_30min | 3 |  |
|  |  | Leaf_tZ-treatment_30min | 3 |  |
|  |  | Leaf_DMSO-treatment_120min | 3 |  |
|  |  | Leaf_tZ-treatment_120min | 3 |  |
| 2. | GSE5167 | 7d_Control | 2 | Jain *et al*., 2009 |
|  |  | 7d_IAA | 2 |  |
|  |  | 7d_BAP | 2 |  |

**References**

**Hirose N, Makita N, Kojima M, Kamada-Nobusada T, Sakakibara H** (2007) Overexpression of a type-A response regulator alters rice morphology and cytokinin metabolism. Plant Cell Physiol **48:** 523-539

**Jain M, Khurana JP** (2009) Transcript profiling reveals diverse roles of auxin-responsive genes during reproductive development and abiotic stress in rice. FEBS J. (in p[ress)

**Jain M, Nijhawan A, Arora R, Agarwal P, Ray S, Sharma P, Kapoor S, Tyagi AK, Khurana JP** (2007) F-box proteins in rice. Genome-wide analysis, classification, temporal and spatial gene expression during panicle and seed development, and regulation by light and abiotic stress. Plant Physiol **143:** 1467-1483

**Li M, Xu W, Yang W, Kong Z, Xue Y** (2007) Genome-wide gene expression profiling reveals conserved and novel molecular functions of the stigma in rice. Plant Physiol **144:** 1797-1812

**Norton GJ, Lou-Hing DE, Meharg AA, Price AH** (2008) Rice-arsenate interactions in hydroponics: whole genome transcriptional analysis. J Exp Bot **59:** 2267-2276

**Ribot C, Hirsch J, Balzergue S, Tharreau D, Notteghem JL, Lebrun MH, Morel JB** (2008) Susceptibility of rice to the blast fungus, *Magnaporthe grisea*. J Plant Physiol **165:** 114-124

**Swarbrick PJ, Huang K, Liu G, Slate J, Press MC, Scholes JD** (2008) Global patterns of gene expression in rice cultivars undergoing a susceptible or resistant interaction with the parasitic plant *Striga hermonthica*. New Phytol **179:** 515-529
