# Supplementary material for: Comprehensive expression analysis suggests overlapping and specific roles of rice glutathione S-transferase genes during development and stress responses
Source: BMC Genomics. 2010 Jan 29;11:73. doi: 10.1186/1471-2164-11-73 (PMC2825235; doi:10.1186/1471-2164-11-73)
Supplement: Additional file 7 — Expression patterns of duplicated GST genes. The average log signal values (from three biological replicates) for each gene in all the samples analyzed is presented on Y-axis. [file 1471-2164-11-73-S7.PPT]

## Slide 1
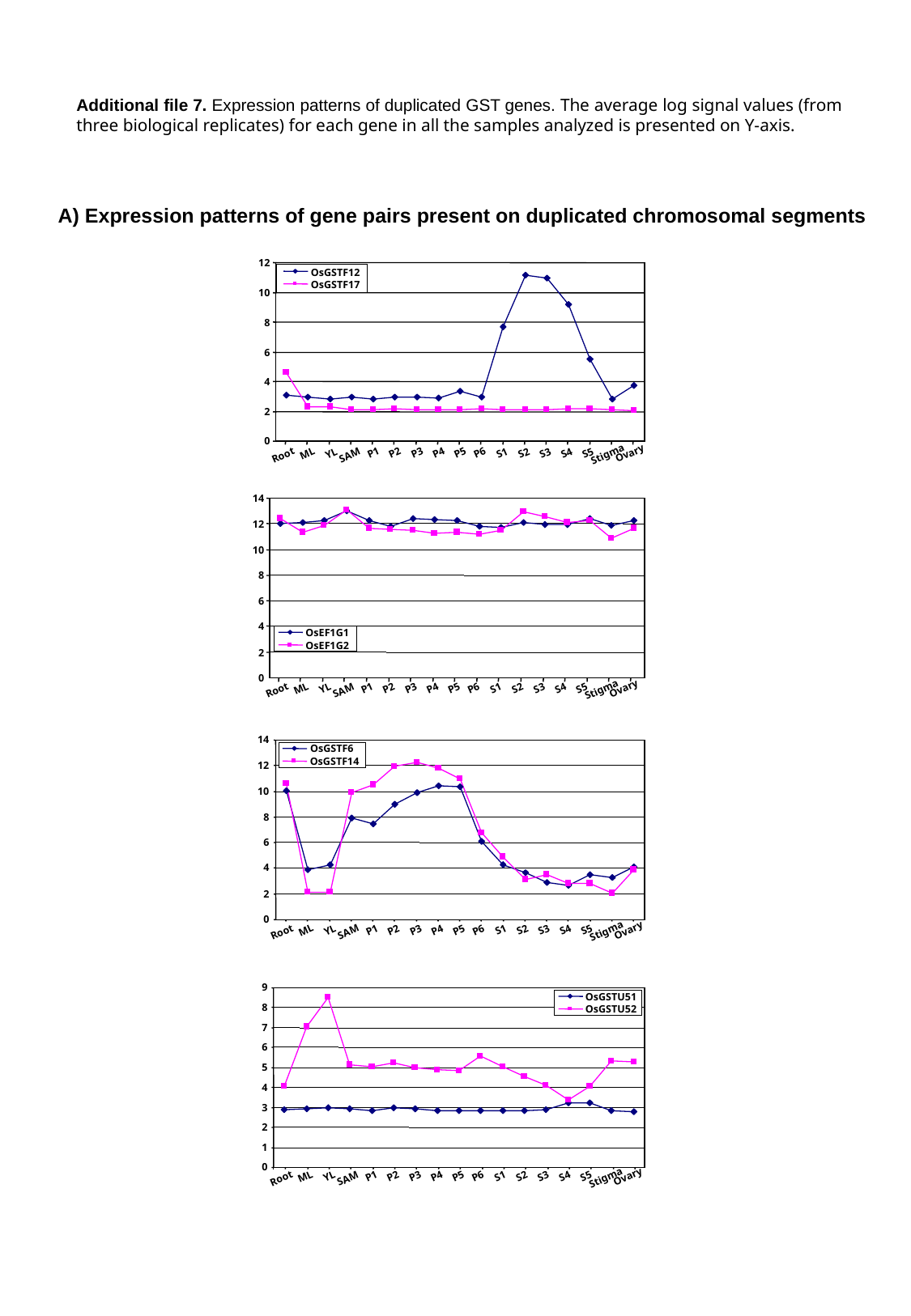

Additional file 7. Expression patterns of duplicated GST genes. The average log signal values (from three biological replicates) for each gene in all the samples analyzed is presented on Y-axis.
A) Expression patterns of gene pairs present on duplicated chromosomal segments
12
OsGSTF12
OsGSTF17
10
8
6
4
2
0
P1
P2
P3
P4
P5
P6
YL
S1
S2
S3
S4
S5
ML
Ovary
Stigma
Root
SAM
14
12
10
8
6
4
OsEF1G1
OsEF1G2
2
0
P1
P2
P3
P4
P5
P6
YL
S1
S2
S3
S4
S5
ML
Ovary
Stigma
Root
SAM
14
OsGSTF6
OsGSTF14
12
10
8
6
4
2
0
P1
P2
P3
P4
P5
P6
YL
S1
S2
S3
S4
S5
ML
Ovary
Stigma
Root
SAM
9
OsGSTU51
OsGSTU52
8
7
6
5
4
3
2
1
0
P1
P2
P3
P4
P5
P6
YL
S1
S2
S3
S4
S5
ML
Ovary
Stigma
Root
SAM

## Slide 2
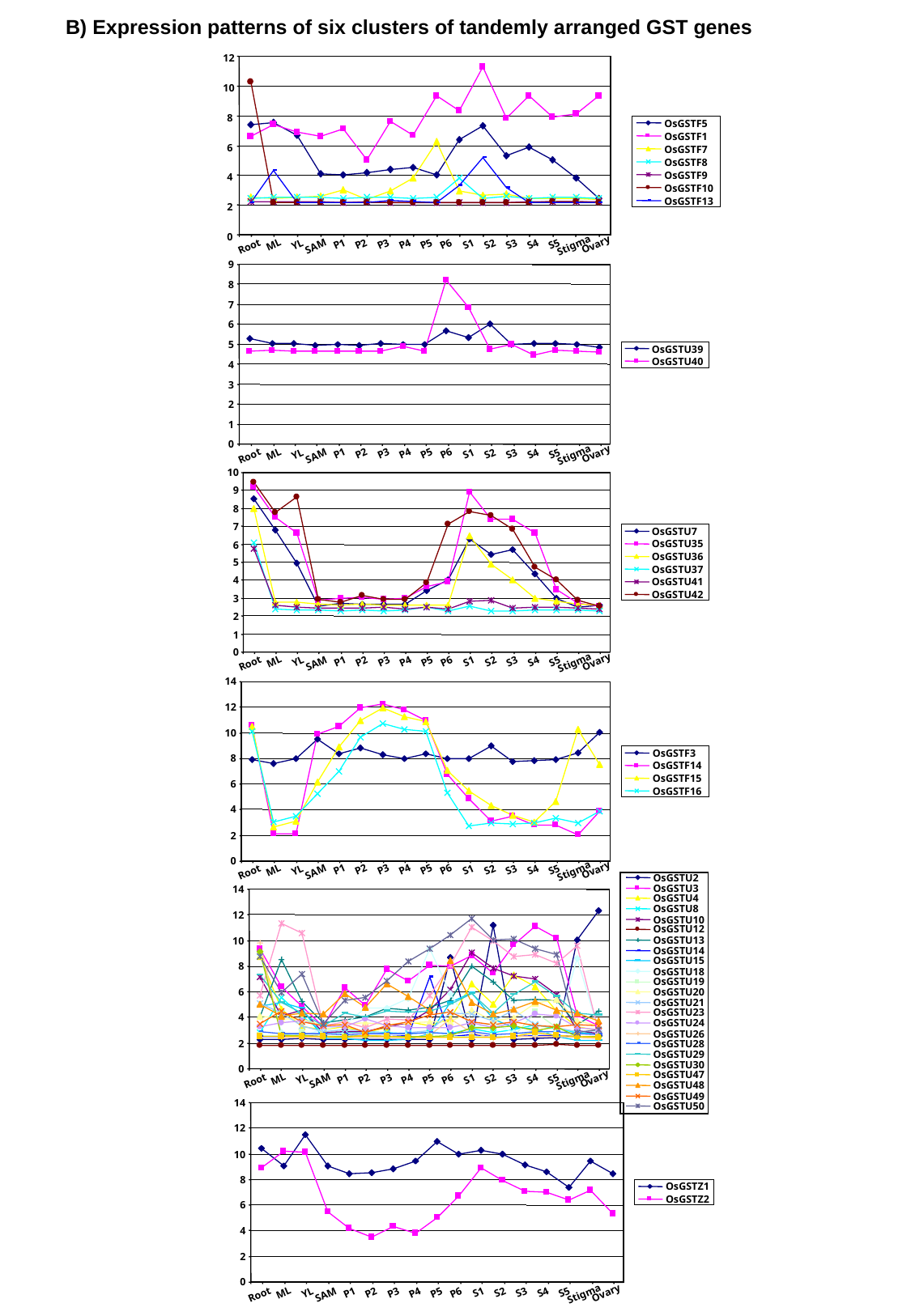

B) Expression patterns of six clusters of tandemly arranged GST genes
12
10
8
OsGSTF5
OsGSTF1
OsGSTF7
OsGSTF8
OsGSTF9
OsGSTF10
OsGSTF13
6
4
2
0
P1
P2
P3
P4
P5
P6
YL
S1
S2
S3
S4
S5
ML
Ovary
Stigma
Root
SAM
9
8
7
6
5
OsGSTU39
OsGSTU40
4
3
2
1
0
P1
P2
P3
P4
P5
P6
YL
S1
S2
S3
S4
S5
ML
Ovary
Stigma
Root
SAM
10
9
8
7
OsGSTU7
OsGSTU35
OsGSTU36
OsGSTU37
OsGSTU41
OsGSTU42
6
5
4
3
2
1
0
P1
P2
P3
P4
P5
P6
YL
S1
S2
S3
S4
S5
ML
Ovary
Stigma
Root
SAM
14
12
10
OsGSTF3
OsGSTF14
OsGSTF15
OsGSTF16
8
6
4
2
0
P1
P2
P3
P4
P5
P6
YL
S1
S2
S3
S4
S5
ML
Ovary
Stigma
Root
SAM
OsGSTU2
OsGSTU3
OsGSTU4
OsGSTU8
OsGSTU10
OsGSTU12
OsGSTU13
OsGSTU14
OsGSTU15
OsGSTU18
OsGSTU19
OsGSTU20
OsGSTU21
OsGSTU23
OsGSTU24
OsGSTU26
OsGSTU28
OsGSTU29
OsGSTU30
OsGSTU47
OsGSTU48
OsGSTU49
OsGSTU50
14
12
10
8
6
4
2
0
P1
P2
P4
P5
YL
P3
P6
S1
S2
S3
S4
S5
ML
Ovary
Stigma
Root
SAM
14
12
10
8
OsGSTZ1
OsGSTZ2
6
4
2
0
P1
P2
P3
P4
P5
P6
YL
S1
S2
S3
S4
S5
ML
Ovary
Stigma
Root
SAM
