# Supplementary material for: Comprehensive expression analysis suggests overlapping and specific roles of rice glutathione S-transferase genes during development and stress responses
Source: BMC Genomics. 2010 Jan 29;11:73. doi: 10.1186/1471-2164-11-73 (PMC2825235; doi:10.1186/1471-2164-11-73)
Supplement: Additional file 8 — Differential expression of rice GST genes in response to plant hormones auxin and cytokinin. (A) Hierarchical clustering of GST genes showing significant differential expression in at least one condition is shown. The fold change values in treated sample as compared to its corresponding mock-treated control sample were used for clustering. The color scale for fold change values is shown at the bottom. IAA, indole-3-acetic acid treatment; BAP, benzyl aminopurine treatment; tZ, trans-zeatin treatment. (B) Real-time PCR analysis of representative GST genes to validate their differential expression during auxin (IAA) and cytokinin (BAP) treatment. The mRNA levels for each gene in different tissue samples were calculated relative to its expression in control seedlings. The error bars represent standard deviation. [file 1471-2164-11-73-S8.PPT]

## Slide 1
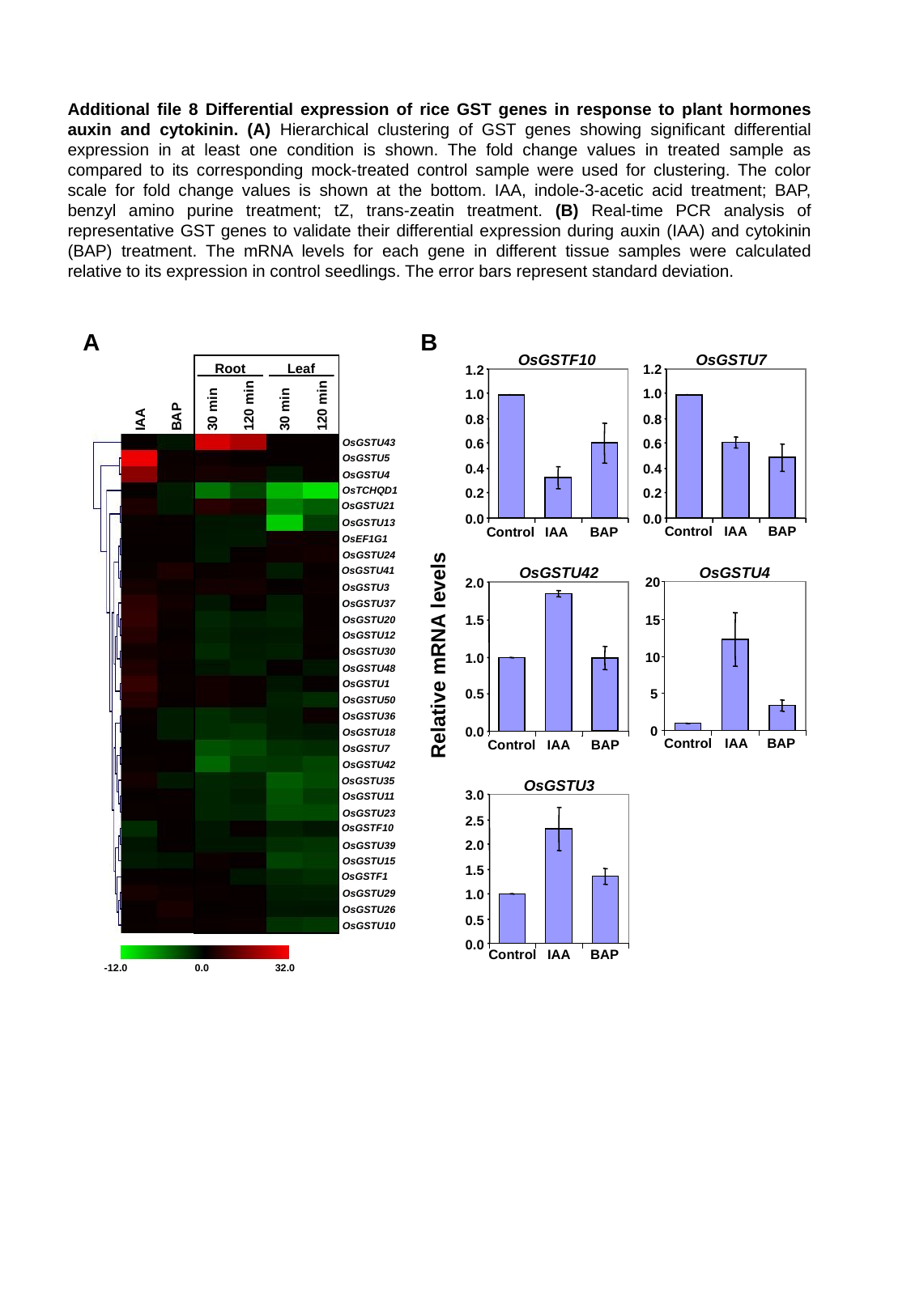

Additional file 8 Differential expression of rice GST genes in response to plant hormones auxin and cytokinin. (A) Hierarchical clustering of GST genes showing significant differential expression in at least one condition is shown. The fold change values in treated sample as compared to its corresponding mock-treated control sample were used for clustering. The color scale for fold change values is shown at the bottom. IAA, indole-3-acetic acid treatment; BAP, benzyl amino purine treatment; tZ, trans-zeatin treatment. (B) Real-time PCR analysis of representative GST genes to validate their differential expression during auxin (IAA) and cytokinin (BAP) treatment. The mRNA levels for each gene in different tissue samples were calculated relative to its expression in control seedlings. The error bars represent standard deviation.
A
B
tZ
Root
Leaf
30 min
120 min
120 min
30 min
IAA
BAP
OsGSTU43
OsGSTU5
OsGSTU4
OsTCHQD1
OsGSTU21
OsGSTU13
OsEF1G1
OsGSTU24
OsGSTU41
OsGSTU3
OsGSTU37
OsGSTU20
OsGSTU12
OsGSTU30
OsGSTU48
OsGSTU1
OsGSTU50
OsGSTU36
OsGSTU18
OsGSTU7
OsGSTU42
OsGSTU35
OsGSTU11
OsGSTU23
OsGSTF10
OsGSTU39
OsGSTU15
OsGSTF1
OsGSTU29
OsGSTU26
OsGSTU10
-12.0
0.0
32.0
OsGSTF10
1.2
1.0
0.8
0.6
0.4
0.2
0.0
Control
IAA
BAP
OsGSTU7
1.2
1.0
0.8
0.6
0.4
0.2
0.0
Control
IAA
BAP
OsGSTU42
2.0
1.5
1.0
0.5
0.0
Control
IAA
BAP
OsGSTU4
20
15
10
5
0
Control
IAA
BAP
Relative mRNA levels
OsGSTU3
3.0
2.5
2.0
1.5
1.0
0.5
0.0
Control
IAA
BAP
