# Supplementary material for: Comprehensive expression analysis suggests overlapping and specific roles of rice glutathione S-transferase genes during development and stress responses
Source: BMC Genomics. 2010 Jan 29;11:73. doi: 10.1186/1471-2164-11-73 (PMC2825235; doi:10.1186/1471-2164-11-73)
Supplement: Additional file 13 — Expression patterns of rice GST genes in various tissues/organs/developmental stages and environmental conditions (various hormone, abiotic stress, arsenate stress and abiotic stress treatments). Hierarchical clustering analysis of 71 GST genes represented on Affymetrix Rice Genome Array is shown. For clustering we used average log signal values for two/three biological replicates of each sample after normalization of the raw data. The color scale for log signal values is shown at the bottom. [file 1471-2164-11-73-S13.PPT]

## Slide 1
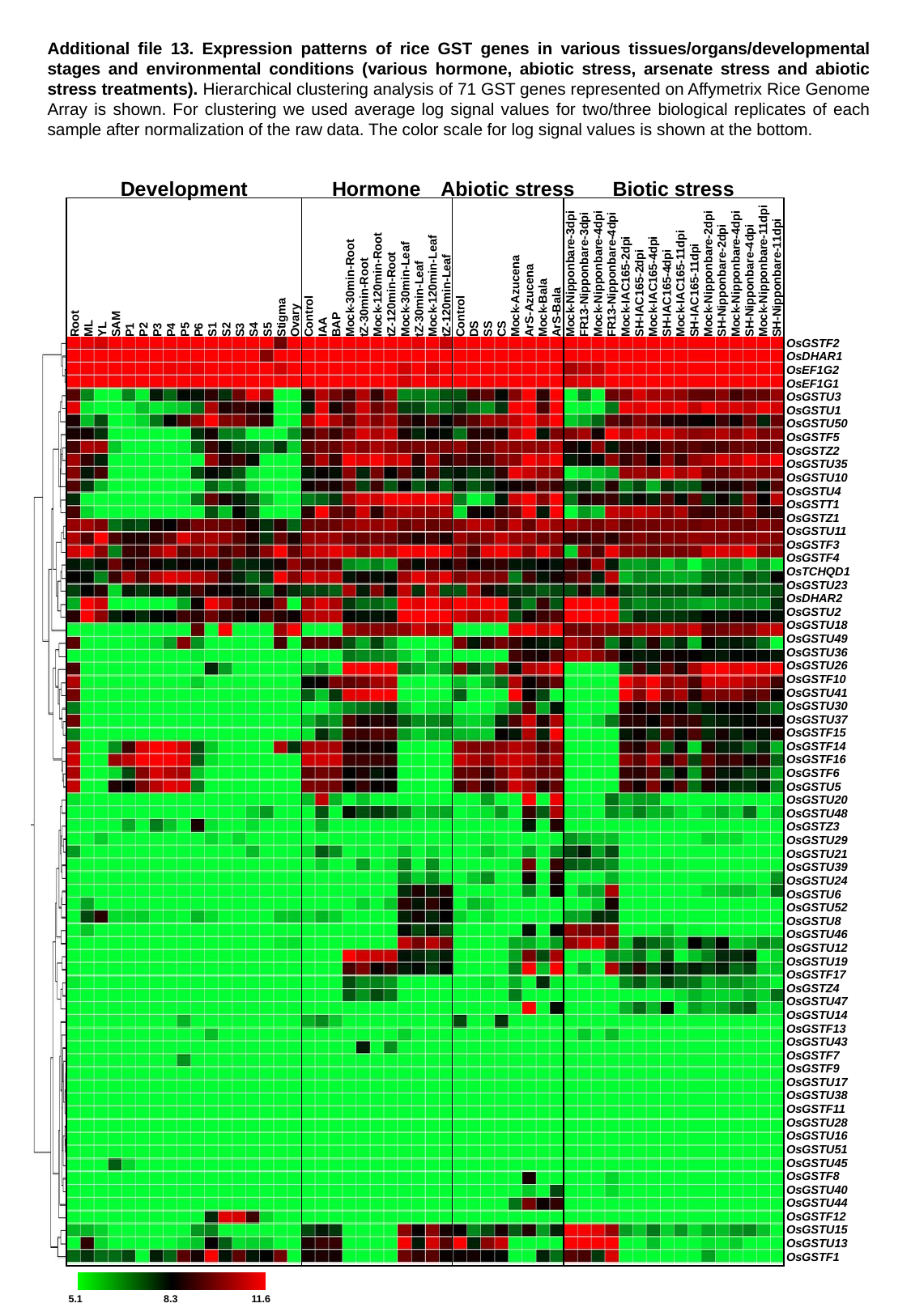

Additional file 13. Expression patterns of rice GST genes in various tissues/organs/developmental stages and environmental conditions (various hormone, abiotic stress, arsenate stress and abiotic stress treatments). Hierarchical clustering analysis of 71 GST genes represented on Affymetrix Rice Genome Array is shown. For clustering we used average log signal values for two/three biological replicates of each sample after normalization of the raw data. The color scale for log signal values is shown at the bottom.
Mock-Nipponbare-11dpi
Mock-Nipponbare-3dpi
FR13-Nipponbare-3dpi
Mock-Nipponbare-4dpi
FR13-Nipponbare-4dpi
Mock-IAC165-2dpi
SH-IAC165-2dpi
Mock-IAC165-4dpi
SH-IAC165-4dpi
Mock-IAC165-11dpi
SH-IAC165-11dpi
Mock-Nipponbare-2dpi
SH-Nipponbare-2dpi
Mock-Nipponbare-4dpi
SH-Nipponbare-4dpi
SH-Nipponbare-11dpi
Mock-120min-Root
Mock-120min-Leaf
Mock-30min-Root
tZ-30min-Root
tZ-120min-Root
Mock-30min-Leaf
tZ-30min-Leaf
tZ-120min-Leaf
Control
DS
SS
CS
Mock-Azucena
ArS-Azucena
Mock-Bala
ArS-Bala
Root
ML
YL
SAM
P1
P2
P3
P4
P5
P6
S1
S2
S3
S4
S5
Stigma
Ovary
Control
IAA
BAP
Development
Hormone
Abiotic stress
Biotic stress
OsGSTF2
OsDHAR1
OsEF1G2
OsEF1G1
OsGSTU3
OsGSTU1
OsGSTU50
OsGSTF5
OsGSTZ2
OsGSTU35
OsGSTU10
OsGSTU4
OsGSTT1
OsGSTZ1
OsGSTU11
OsGSTF3
OsGSTF4
OsTCHQD1
OsGSTU23
OsDHAR2
OsGSTU2
OsGSTU18
OsGSTU49
OsGSTU36
OsGSTU26
OsGSTF10
OsGSTU41
OsGSTU30
OsGSTU37
OsGSTF15
OsGSTF14
OsGSTF16
OsGSTF6
OsGSTU5
OsGSTU20
OsGSTU48
OsGSTZ3
OsGSTU29
OsGSTU21
OsGSTU39
OsGSTU24
OsGSTU6
OsGSTU52
OsGSTU8
OsGSTU46
OsGSTU12
OsGSTU19
OsGSTF17
OsGSTZ4
OsGSTU47
OsGSTU14
OsGSTF13
OsGSTU43
OsGSTF7
OsGSTF9
OsGSTU17
OsGSTU38
OsGSTF11
OsGSTU28
OsGSTU16
OsGSTU51
OsGSTU45
OsGSTF8
OsGSTU40
OsGSTU44
OsGSTF12
OsGSTU15
OsGSTU13
OsGSTF1
5.1
8.3
11.6
