# Supplementary material for: Comprehensive expression analysis suggests overlapping and specific roles of rice glutathione S-transferase genes during development and stress responses
Source: BMC Genomics. 2010 Jan 29;11:73. doi: 10.1186/1471-2164-11-73 (PMC2825235; doi:10.1186/1471-2164-11-73)
Supplement: Additional file 14 — Primer sequences used for real-time PCR analysis. [file 1471-2164-11-73-S14.DOC]

**Additional file 14.** Primer sequences used for real-time PCR analysis.

| **Gene name** | **Locus** | **Primer sequence** |
| --- | --- | --- |
| *OsGSTF5* | Os01g27210 | 5'- CCG TTC GGT GAG ATT CCA GTA -3' |
|  |  | 5'- CGA TTG CTC GCG ATT GAT AG -3' |
| *OsGSTF10* | Os01g27390 | 5'- ATC TCC CGT AAC CCC TTT GG -3' |
|  |  | 5'- CAG AGT CAG GTC GCC ATC CT -3' |
| *OsGSTF12* | Os01g27630 | 5’- CCC ATG TCC GTG GTC ATC TAC -3’ |
|  |  | 5’- GGT TTG TCC ACC GAA ATA CAC A -3’ |
| *OsGSTU3* | Os10g38501 | 5'- GAC GAC AAG CTG TTG AAG TCA TG -3' |
|  |  | 5'- GCC TTC TCC TGC TCC GTC TT -3' |
| *OsGSTU4* | Os10g38495 | 5'- CTA CGT CGA CGA CAA GTT CGT T -3' |
|  |  | 5'- TCC TCC GTC TTG CCT CTG AA -3' |
| *OsGSTU5* | Os09g20220 | 5’- AGG TTC TGG GCG GAC TAC GT -3’ |
|  |  | 5’- GAG CTT CCA GAG GCG TGT CT -3’ |
| *OsGSTU7* | Os01g72120 | 5'- ATG GAG TGC TTG CCG GAT AG -3' |
|  |  | 5'- TCT GCT TGA TCC CGG TGA AG -3' |
| *OsGSTU39* | Os01g49720 | 5’- GCG CGT TTC TGG ACC AAC TA -3’ |
|  |  | 5’- TCG ACC TGA ACA GCA CTT TCC -3’ |
| *OsGSTU40* | Os01g49710 | 5'- AGG GCC TGA GCT ACG AGT ACA T -3' |
|  |  | 5'- TTG AGC AGG AGC TCG CTC TT -3' |
| *OsGSTU42* | Os01g72170 | 5'- GCT TCG CGG CGG AGA T -3' |
|  |  | 5'- AAG AAT CGC CTC CCC TTG AG -3' |
| *OsGSTZ2* | Os12g10730 | 5’- CAA ATT TGG CAA GAC TCC ATG A -3’ |
|  |  | 5’- GAA GTG CTG CCT GAA ATG CA -3’ |
| *OsTCHQD1* | Os04g35660 | 5'-CTC ACT CAC ACC CCG ATC AA |
|  |  | 5'-AGC ACT CGC CGT ATG AAC TTG |
